# Assessment of the potential for genomic selection to improve resistance to fusarium stalk rot in maize

**DOI:** 10.3389/fpls.2025.1631408

**Published:** 2025-09-23

**Authors:** Hirenallur Chandappa Lohithaswa, B. M. Showkath Babu, Muntagodu Shreekanth Sowmya, Santhosh Kumari Banakar, Nanjundappa Mallikarjuna, Ganiga Jadesha, Mallana Gowdra Mallikarjuna, D. C. Balasundara, Pandravada Anand

**Affiliations:** ^1^ Department of Genetics and Plant Breeding, University of Agricultural Sciences, Gandhi Krishi Vignana Kendra (GKVK), Bengaluru, Karnataka, India; ^2^ All India Co-ordinated Research Project (AICRP) on Maize, Zonal Agricultural Research Station, V. C. Farm, Mandya, Karnataka, India; ^3^ Maize Genetics Laboratory, Division of Genetics, Indian Agriculture Research Institute, New Delhi, India; ^4^ Corteva Agriscience Research Farm, Chikkaballapur, Karnataka, India

**Keywords:** maize, fusarium stalk rot (FSR), doubled haploids, GEBVS, genomic prediction, genomic selection

## Abstract

Fusarium stalk rot (FSR), caused by *Fusarium verticilliodes*, is a serious disease in maize. Resistance to FSR is complexly inherited. Thus, an investigation was carried out to predict and validate the genomic estimated breeding values (GEBVs) for FSR resistance. Three doubled haploid (DH) populations induced from F_1_ and F_2_ of the cross VL1043 × CM212 and F_2_ of the cross VL121096 × CM202 were used in the current study. Six different parametric models (Genomic-Best Linear Unbiased Predictors (GBLUP), BayesA, BayesB, BayesC, Bayesian least absolute shrinkage and selection operator (BLASSO), and Bayesian Ridge Regression (BRR)) were employed to estimate the prediction accuracy. Further, the accuracy of predicted genomic estimated breeding value (GEBV) for FSR resistance was assessed using five-fold cross-validation and independent validation. The training population (TP) size and marker density were optimized by considering different proportions of training set (TS) and validation set (VS) and varying marker density from 40 to 100%. The estimates of descriptive statistics and genetic variability parameters, which include mean, standardized range, genetic variance, phenotypic and genotypic coefficients of variations, broad sense heritability, and genetic advance as per cent mean (GAM), were relatively higher in DH F_2_s than those in DH F_1_s. Prediction accuracies displayed an increasing trend with an increase in the proportion of training set size and marker density in all three DH populations. The TS:VS proportion of 75:25 in DH F_1_ (VL1043 × CM212) and DH F_2_ (VL121096 × CM202), and 80:20 in DH F_2_ of VL1043 × CM212 resulted in greater prediction accuracy than other TS:VS proportions. Study of linkage disequilibrium (LD) decay pattern across all the populations indicated that the number of markers employed were sufficient to conduct a genomic prediction (GP) study in two DH F_2_ populations of crosses VL1043 × CM212 and VL121096 × CM202. Prediction accuracies of 0.24 and 0.17 were recorded for FSR resistance in independent validation when DH F_2_ of cross VL121096 × CM202 was used for validation and DH F_1_ and DH F_2_s from the cross VL1043 × CM212 as training sets. A significant positive correlation of FSR resistance between the DHs selected based on their GEBVs and those selected based on test cross performance indicated the efficiency of genomic prediction models.

## 1 Introduction

Maize (*Zea mays* L.) is considered vital to the world’s agriculture and is a treasured resource that provides food, fodder, and industrial raw materials (Agrawal et al., 2018). The annual growth rate of maize production (1.6%) in the current climate change years is insufficient to meet the global demands projected for 2050 (Ray et al., 2013; Erenstein et al., 2022). Maize is affected by as many as 130 pests and about 110 diseases globally (Ray et al., 2013). The diseases of maize include seedling blights, foliar diseases, downy mildews, fusarium stalk rots, wilts, rusts, smuts, and ear rots. Among all maize diseases, post-flowering stalk rot (PFSR) is considered the world’s most destructive disease in recent years and is widely distributed in all maize agro-ecologies (Showkath Babu et al., 2020). PFSR is a complex disease caused by many fungi involved in decaying the pith, resulting in pre-mature wilting of the plants. Pathogens such as *Fusarium verticilloides* (Fusarium stalk rot), *Macrophomina phaseolina* (Charcoal rot) and *Harpophora maydis* (Late wilt) are commonly associated with PFSR. Fusarium stalk rot (FSR) caused by the pathogen *Fusarium verticilioides* (Saccardo) Nirenberg (formerly called *Fusarium moniliforme*) (Seifert et al., 2003) is considered to be a serious threat to maize cultivation in the world including India. In India, the disease is prevalent in most maize-growing areas, where water stress occurs after the flowering stage (Singh et al., 2012). The incidence of FSR after the flowering stage and before physiological maturity results in reduced yields as affected plants die prematurely, producing light-weight ears with poorly filled kernels. Plants infected with stalk rot lodge easily, which makes harvesting difficult and ears are left in the field while harvesting. The disease incidence ranges from 10 to 42% (Desai et al., 1992; Kumar et al., 1998; Harlapur et al., 2002) in major maize-growing areas. Additionally, the FSR can cause a reduction of 18.70% in cob weight and 11.20% in 100 grain weight in the infected plants (Cook, 1978).

Among the various strategies for managing FSR disease in maize, breeding for resistance is the most practical, cost-effective and eco-friendly approach (Jeevan et al., 2020; Showkath Babu et al., 2020). The quantitative nature of FSR resistance (Szoke et al., 2007; Khokhar et al., 2014; Archana et al., 2019; Showkath Babu et al., 2020, 2024) has resulted in a rather slow and limited genetic gain per unit of time through conventional plant breeding (Enrico Pè et al., 1993; Yang et al., 2004; Mir et al., 2018). The difficulties of conventional breeding favoured the development and utilization of genomic tools in breeding complex traits like FSR resistance.

Marker assisted selection has proved effective to improve only traits controlled by one or a few large-effect loci. However, the FSR resistance is controlled by both large and small effect quantitative trait loci (QTLs) (Rashid et al., 2022; Showkath Babu et al., 2024). Thus, capturing both large and small effect QTLs is crucial for developing improved maize hybrids with enhanced FSR resistance (Dekkers and Hospital, 2002). At this juncture, the genomic selection (GS) was proposed to capture both small and large effect QTLs (Meuwissen et al., 2001; Bernardo and Yu, 2007; Mayor and Bernardo, 2009).

Genomic selection is defined as the selection of genotyped-only breeding population (BP) individuals based on their GEBVs predicted using marker effects estimated by fitting statistical models calibrated in both genotyped and phenotyped training population (TP) (Meuwissen et al., 2001). Genomic selection models work well in terms of high prediction accuracy if the individuals of the training and breeding population are related. A diverse training population, including both related and unrelated genotypes, can lead to more broadly applicable prediction models. Individuals from the same family or biparental cross can also be used as both the training and breeding populations although population structure significantly impacts genomic prediction accuracy (Riedelsheimer et al., 2013; Zhang et al., 2015, 2017; Schopp et al., 2017; Brauner et al., 2020). Genomic prediction in biparental populations, has been proved to be a very effective scheme for identifying superior lines in plant breeding programs. This approach powers the strong genetic relationship between the training and prediction sets, which maximizes linkage disequilibrium between markers and quantitative trait loci (QTL). It allows for accurate prediction of traits even with limited marker density and relatively small training populations (Riedelsheimer et al., 2013). To perform GS, TP is used to train or calibrate a statistical model to estimate the marker effects. The calibrated/trained statistical model is then used to predict GEBVs of non-phenotyped but genotyped-only BP individuals. The GEBVs of individuals of the breeding population are predicted as the sum of the effects associated with all marker alleles irrespective of whether they are linked or unlinked to QTLs controlling target traits. Thus, the GS is described as MAS without QTL mapping (Bernardo and Yu, 2007; Mayor and Bernardo, 2009).

The effectiveness of GS depends on the accuracy of predicted GEBVs, which in turn depends on the training population (TP) composition and its size as well as its genetic relatedness with the BP (Wang et al., 2017). Other factors influencing the accuracy of GEBV are the statistical model used for prediction, the density of markers and the heritability of target traits (Bernardo and Yu, 2007; Bernardo, 2009; Goddard and Meuwissen, 2010; Massman et al., 2013; Song et al., 2018).

The use of genomic tools in combination with doubled haploids (DH) technology, which results in the completely homozygous lines in the quickest possible time has been suggested to enhance the genetic gain per breeding cycle and unit time. The DH offer several advantages over mapping populations, through the fastest attainment of complete homozygosity, lack of residual heterozygosity and accurate phenotyping compared to families in early segregating generations (F_3_ or F_4_) (Yan et al., 2009). High genetic variance in DH lines is directly proportional to response to selection (Stich et al., 2005; Bordes et al., 2007; Mayor and Bernardo, 2009). The DH lines also offer opportunities for improving selection gain and increasing the precision and accuracy of quantification of genetic × environment interactions for identifying the genomic regions for key traits (Mansur et al., 1996). DH lines can be induced from F_1_ or F_2_ as base populations, which depends upon various factors including time needed to create DH populations, amount of recombination and ability to select superior plants before haploid induction (Bernardo, 2009; Showkath Babu et al., 2023).

The use of the most appropriate filial generations (F_1_/F_2_) to induce DH and optimized parameters of genomic prediction is expected to result in rapid and greater genetic gain for target traits. Thus, the current investigation was framed to predict and validate the GEBVs for FSR resistance in DH populations induced from F_1_ and F_2_ populations and to optimize the size of the training population and marker density to be used to attain the highest prediction accuracy.

## 2 Materials and methods

### 2.1 Phenotypic data

#### 2.1.1 Basic genetic material

The primary material for the study consisted of two highly susceptible inbreds namely VL1043 (CLQRCYQ59-B*4) and VL121096 (NEI9008-B*12) and two moderately resistant inbreds CM212 (USA/ACC No.2132 (Alm)-3-2-f-#-13-#-⊗-bulk) and CM202 (C121, Early). These inbred lines were procured from the International Maize and Wheat Improvement Center (CIMMYT), Asia Centre for Maize, Hyderabad. The inbred lines were selected based on the previous year’s disease reaction from artificial disease screening data against FSR (Archana et al., 2021.

#### 2.1.2 Development of DH lines

The methodology for material development was described in the earlier study by Showkath Babu et al. (2023). The 336 DHF_1_ lines derived from the cross VL1043 × CM212 along with parents as checks were screened for their response to FSR by artificial inoculation during the winter season of 2018-19, the rainy season of 2019 and the winter season of 2019 - 20. Similarly, the DH lines (280 and 94) derived from F_2_ plants of the crosses VL1043 × CM212 and VL121096 × CM202 were phenotyped during the rainy season of 2019 and winter season of 2019-20. Each DH line was planted in a row of 2 m in length with an inter-row spacing of 0.6 m and inter-plant spacing of 0.2 m at the College of Agriculture, V.C. Farm, Mandya (Latitude: 12°31’21.94” N; Longitude: 76°54’24.16” E; Altitude: 729 meters above mean sea level), in an augmented design (Federer, 1961) with checks replicated twice within each block.

#### 2.1.3 Phenotyping DH lines for responses to FSR

The procedure for isolation and multiplication of *Fusarium verticilloides* pathogen was followed as given by Hooda et al. (2018) and Showkath Babu et al. (2023). To all the plants, established in the field 2 ml of the inoculum containing 1×10^6^ spores/ml was injected diagonally using the syringe after pricking and making a 2 cm hole with the help of a jabber to the second internode from the base at 65 and 75 days after sowing to ensure effective and uniform disease incidence. After inoculation, irrigation was withheld for four days to enable proper uptake of inoculum by the plants and all the recommended production practices were followed except the spray of fungicides to maintain the plants after inoculation. Disease screening was carried out following the procedure developed by Hooda et al. (2018).

#### 2.1.4 Sampling and data recording

For disease phenotyping, the stalks were split open before drying, *i.e.*, 30 days after inoculation. Disease severity and intensity were recorded on individual plants of each line using a 1 – 9 rating disease scale (**Table 1**) in all the seasons (**Supplementary Tables 1a-c**). The scoring pattern was based on the spread of discoloration inside the maize stalks from the point of inoculation (Payak and Sharma, 1983). Higher extent of discoloration implies higher rating of FSR incidence.

**Table 1:** Disease rating scale for Fusarium stalk rot (Payak and Sharma 1983).

Disease score	Symptoms	Disease reaction
1	Healthy or slight discolouration at the site of inoculation	Highly resistant
2	Up to 50% of the inoculated internode is discoloured	Resistant
3	51-75% of the inoculated internode is discoloured	Moderately resistant
4	76-100% of the inoculated internode is discoloured	Moderately susceptible
5	Less than 50% discolouration of the adjacent internode	Susceptible
6	More than 50% discolouration of the adjacent internode	Highly susceptible
7	Discolouration of three internodes	Highly susceptible
8	Discolouration of four internodes	Highly susceptible
9	Discolouration of five or more internodes and premature death of plant	Highly susceptible

#### 2.1.5 Phenotypic data analysis

The disease score obtained on 336 DHF_1_s and 280 DHF_2_s derived from the cross VL1043 × CM212, and 94 DHF_2_s from the cross VL121096 × CM202 for individual seasons were subjected statistical analyses using Augmented design. Each block within a season contained unreplicated test entries and a set of replicated checks. This structure was used to efficiently evaluate the large number of DH lines with limited replication. Given the distinct replication structure and analytical objectives of the test entries and the checks, two complementary statistical approaches were employed.

##### 2.1.5.1 Analysis of variance

Disease scores of each of the DH lines in individual seasons were analysed using augmentedRCBD package (Aravind et al., 2023) of ‘R’ software version 4.3.1. Further, pooled augmented analysis was done to account for the variability and environmental influence, the linear model for the same is given below (Merrick and Carter, 2021) in **Equation 1**.


(1) $$Y_{ijkl}\;=\mu \;\;+\;Block_{i}\;+\;Check_{j\;}\;+\;Env_{l}+\;Block_{i}\times Env_{l}\;+\;Check_{j\;}\times Env_{l}\;+\varepsilon_{ijk}$$

Where,

$Y_{ij}$ - phenotypic value of the $i^{th}$ block and $j^{th}$ check in the $k^{th}$ environment

μ- overall mean

$Block_{i}$ - random effect of the $i^{th}$ block with the distribution Block ∼N (0, ${\text{σ}}_{\text{Block}}^{2}$ )

$Check_{j\;}$ - fixed effect of the $j^{th}$ replicated check cultivar

$Env_{l}$ - random effect of the $l^{th}$ environment with the distribution Env ∼*N* (0, ${\text{σ}}_{\text{Env}}^{2}$ ) and

$\varepsilon_{ijk}$ - residual errors with a random normal distribution of ε∼*N* (0, $\sigma_{\varepsilon }^{2}$ )

##### 2.1.5.2 Estimation of genetic variability parameters

Phenotypic coefficient of variability (PCV), Genotypic co-efficient of variability (GCV), heritability (broad sense) and genetic advance and genetic advance as per cent of mean were estimated as follows,

###### 2.1.5.2.1 Phenotypic coefficient of variation (PCV)

The formula for computation of PCV is given in **Equation 2**.


(2) $$\text{PCV}(\%)=\frac{\sqrt{\sigma_{p}^{2}}}{\bar{x}}\;\times \;100$$

Where,

$\sigma_{\text{p}}^{2}$ - Phenotypic variance 

$\bar{X}$ - Overall mean

###### 2.1.5.2.2 Genotypic coefficient of variation (GCV)

The formula for computation of GCV is given in **Equation 3**.


(3) $$\text{GCV}(\%)=\frac{\sqrt{\sigma_{g}^{2}}}{\bar{x}}\;\times \;100$$

Where, $\sigma_{\text{g}}^{2}$ - Genotypic variance

$\bar{X}$ - Overall mean

PCV and GCV were classified as low, moderate and high as suggested by Robinson et al. (1949).

Broad sense heritability (H) was estimated using the following formula given by Hanson et al. (1956) as given in **Equation 4**.


(4) $$\text{H}(\%)=\frac{\sigma_{g}^{2}}{\sigma_{p}^{2}}\times 100$$

Where, $\sigma_{g}^{2}$ = Genotypic variance

$\sigma_{p}^{2}$ = Phenotypic variance

Expected Genetic advance (GA) was figured by the following formula given by Johnson et al. (1955) as given in **Equation 5**.


(5) $$\text{GA}=k\;\times \;h_{b}^{2}\times \;\sqrt{\sigma_{p}^{2}}$$

Where, k = selection differential (2.06) at 5% selection intensity and $\surd \sigma_{p}^{2}$ = phenotypic standard deviation

The expected genetic advance as a per cent of the mean was estimated as given in **Equation 6**.


(6) $$\text{GAM}=\frac{\text{GA}}{\text{μ}}\times 100$$

Where GA is the genetic advance and μ is the general mean.

##### 2.1.5.3 BLUEs and BLUPs calculation

###### 2.1.5.3.1 BLUEs estimation

The best linear unbiased estimates (BLUEs) for the unreplicated DHF_1_ and DHF_2_ populations were obtained using a mixed linear model present in lme4 package (Bates et al., 2015) in R software version 4.4.1. the genotypes and seasons were treated as fixed effects and blocks nested within season was modeled as random effect to account for the environmental variation. The genotype × season interaction was also included in the model as a fixed effect to account for the differential genotypic responses across seasons. The model used for the BLUEs estimation is given below in **Equation 7**..


(7) $$Y_{ijk}\;=\mu \;+\;S_{i}\;+\;B_{j\;(i)}\;+\;G_{k}+\;(G_{k}\times S_{i})\;+\;\varepsilon_{ijk}$$

Where, $Y_{ijk}$ is the disease score, μ  is the overall mean (fixed), $S_{i}$ is the fixed effect of season i, $B_{j(i)}$ is the random effect of block j within season i, $G_{k}$ is the fixed effect of genotype k, $G_{k}\times S_{i}$ is the fixed effect of genotype by season interaction, and $\varepsilon_{ijk}$ is the residual error.

###### 2.1.5.3.2 BLUPs estimation

For the unreplicated DHF_1_ and DHF_2_s, best linear unbiased predictors (BLUPs) were estimated across seasons using a linear model implemented in the package lme4 (Bates et al., 2015) of R version 4.4.1. BLUPs maximize the correlation between the predicted and true genetic values and account for the variance components and interaction effects, improving accuracy (Beavis and Mahama, 2023).

The linear model used for across seasons BLUPs estimation is given below in **Equation 8**.


(8) $$Y_{ijk}\;=\mu \;+\;S_{i}\;+\;B_{j\;(i)}\;+\;G_{k}+\;(G_{k}\times S_{i})\;+\;\varepsilon_{ijk}$$

Where, $Y_{ijk}$ is the disease score, μ  is the overall mean, $S_{i}$ is the fixed effect of season i, $B_{j(i)}$ is the random effect of block j within season i, $G_{k}$ is the random effect of genotype k, $G_{k}\times S_{i}$ is the random effect of genotype by season interaction, and $\epsilon_{ijk}$ is the residual error.

BLUPs estimate both fixed and random effects, whereas BLUEs estimate only fixed effects and do not shrink estimates toward the mean. This lack of shrinkage can lead to overestimation, particularly in unbalanced datasets or those with small sample sizes (Henderson, 1975). However, the correlation between BLUEs and BLUPs was high (>0.90) across all three DH populations, and genotype rankings remained largely unchanged. Therefore, BLUP-based genomic prediction was carried out in this study.

The choice to implement two separate statistical approaches was based on the design structure and analytical objectives. The replicated check cultivars allowed the use of traditional ANOVA to evaluate standard genotype performance and environmental variability. In contrast, the unreplicated DH lines required a mixed model to accurately predict genotypic values while accounting for random effects and genotype × season interaction.

##### 2.1.5.4 Genotyping of doubled haploid lines

Seeds of three DH populations, *i.e.* F_1_ and F_2_ induced (VL1043 × CM212) and F_2_ induced (VL121096 × CM202) were subjected to genotyping using Corteva AgriScience Proprietary-Single Nucleotide Polymorphisms (SNPs) markers through Illumina Infinium XT assay. Polymorphic markers between parents were chosen for genotyping the DH progenies. From the 2000 Corteva proprietary markers, a total of 198, 199 and 193 SNPs (**Supplementary Tables 2a–c**) remained after filtering for call rate of > 0.90, minor allelic frequency (MAF) > 0.05 and heterozygosity of > 0.1, in DHF_1_, DHF_2_ and DHF_2_ progenies of the crosses VL1043 × CM212 and VL121096 × CM202, respectively were used in the current study.

##### 2.1.5.5 Prediction and validation of genomic estimated breeding values (GEBVs) for FSR resistance

### 2.2 Prediction models

The material consisted of 336 DHF_1_s and 280 DHF_2_s derived from cross VL1043 × CM212 and 94 DHF_2_ lines from the cross VL121096 × CM202. Adjusted average FSR disease scores from individual seasons were considered and pooled to get the disease scores across the seasons and this phenotypic data along with the genotypic data were used in all the prediction studies. Six parametric models that include Genomic BLUP (VanRaden, 2008), BayesA (Meuwissen et al., 2001), BayesB (Spiegelhalter et al., 2002), BayesC (Spiegelhalter et al., 2002), Bayesian ridge regression (BRR) (Shi et al., 2016) and Bayesian LASSO (Park and Casella, 2008) models (Perez et al., 2010) were used to estimate the marker effects using *BGLR* () function with 1,00,000 iterations and 20,000 burnins in each fold of a five fold cross validation in BGLR package (Perez and de Los Campos, 2014) of ‘R’ software version 4.3.1.

#### 2.2.1 GBLUP

It is the technique that utilizes the genomic relationship among individuals to estimate the genetic merit of an individual. It is based on mixed model equations (MME) and best linear unbiased predictors (BLUPs). This model assumes additive genetic effects follow a normal distribution. The GBLUP model when all the markers are considered random is represented as in **Equation 9**.


(9) $$y\;=\;\mu 1_{n}\;+\;Zg\;+\;e$$

Where y is the value of the trait, μ is the overall mean, g is a vector of additive genetic effects estimated using markers considered random, Z is the design matrix associating g with response variables, g is the genomic relationship matrix, and e is a residual effect, with the following distributions. The G matrix was computed by following VanRaden’s (2008) method. The formula for the same is given in **Equation 10**.


(10) $$G\;=\;\frac{zz^{\prime }}{2\sum p_{j}(1\;-p_{j})}$$

Where, G is the genomic relationship matrix, $zz^{\prime }$ measures the genomic covariance between the individuals and $p_{j}$ is the allelic frequency.


$$\text{G}∼\text{N}(0,{\text{ Gσ}}_{\text{g}}^{2}),$$

$$\text{e}∼\text{N}(0,{\text{ Rσ}}_{\text{e}}^{2}),$$

This model works best for polygenic traits with small individual marker effects.

#### 2.2.2 Bayesian alphabet models

These methods involve two major steps, i) estimating marker effects using the genotypic and phenotypic data of the training set utilizing different models and ii) using the estimated marker effects to get the GEBVs of the individuals in the validating set or breeding population. All these Bayesian statistical models differ in their prior assumptions of marker effects. The statistical representation of BayesA, BayesB, BayesC, BLASSO, and Bayesian Ridge Regression (BRR) considering all the marker effects as fixed is, given in the **Equation 11**, below.


(11) $$y\;=\;\mu 1_{n}\;+\;X\beta +\;e$$

Where, y = value of the trait, μ = overall mean, X = genotypic matrix containing values 0, 1, and 2, β = random vector of marker effects and e = random vector of residuals with e ~ N (0, I_n_ σ^2e^).

The Bayesian alphabets differ in their prior distributions of variances of marker effects (β). BRR, and BLASSO assume a normal distribution, while BayesA, BayesB, and BayesC assume a scaled t-student distribution, spike-lab with the scaled t-student and spike-lab with the normal distribution for the variances of marker effects, respectively.

Further, the GEBVs were calculated using the formula, as given in **Equation 12**, below.


(12) $${\hat{g}}_{i}\;=\sum_{i}^{n}Z_{ij}\;{\hat{m}}_{i}\;$$

Where, ‘*m*’ is the vector of random marker effects, ‘*Z* ‘ is the incidence matrix m, ‘*i*’ is the specific allele of the i^th^ SNP marker on individual ‘*j*’ and it denotes the allele or genotype score for a given SNP in an individual and ‘*n*’ is the total number of markers.

The predicted GEBVs were cross-validated using five-fold cross-validation, wherein the entire population was divided into five folds. The prediction accuracy was estimated by considering 1,00,000 iterations and 20,000 burnins in each fold of a five-fold cross-validation.

##### 2.2.2.1 Computation of prediction error

Random mean of squared errors (RMSE) was computed to estimate the prediction error between the observed and estimated prediction abilities after each round. RMSE is a commonly employed metric to summarize the error (Hastie et al., 2009). It gives a single measure that reflects the average magnitude of the prediction errors across the folds, penalizing larger errors more heavily. It is mainly used in comparing the predictive performance of different models.

The formula for estimating the predictive error is given in **Equation 13**.


(13) $$\text{RMSE}=\sqrt{\frac{1}{n}}\sum_{i=1}^{n}{\left(y_{i}-{\hat{y}}_{i}\right)}^{2}$$

Where $y_{i}$ is the observed value, ${\hat{y}}_{i}$ is the predicted value, and n is the total number of observations across all the folds. Lower RMSE values indicate better predictive accuracy.

### 2.3 Evaluation of the accuracy of predicted GEBVs of individuals of VS

The GS effectiveness depends on the accuracy of predicted GEBVs and it is quantified as the correlation ( $r\hat{g}g$ ) between predicted GEBVs ( $\hat{g}$ ) and true breeding values (g). As true breeding values are not known a prior, correlation ( $r\hat{y}y$ ) between predicted GEBVs ( $\hat{y}$ ) and observed phenotype values (y) is the estimated predictive ability (PA). The GEBVs prediction accuracy is then estimated as the ratio of PA to the square root of heritability (h) (Dekkers, 2007). Thus, the accuracy of predicted GEBVs was computed as in the **Equation 14**, given below.


(14) $$r_{\hat{g}g}\;=\frac{r_{\hat{y}y}}{h}$$

### 2.4 Comparing strategies of training population size and marker density

**Figure 1:**
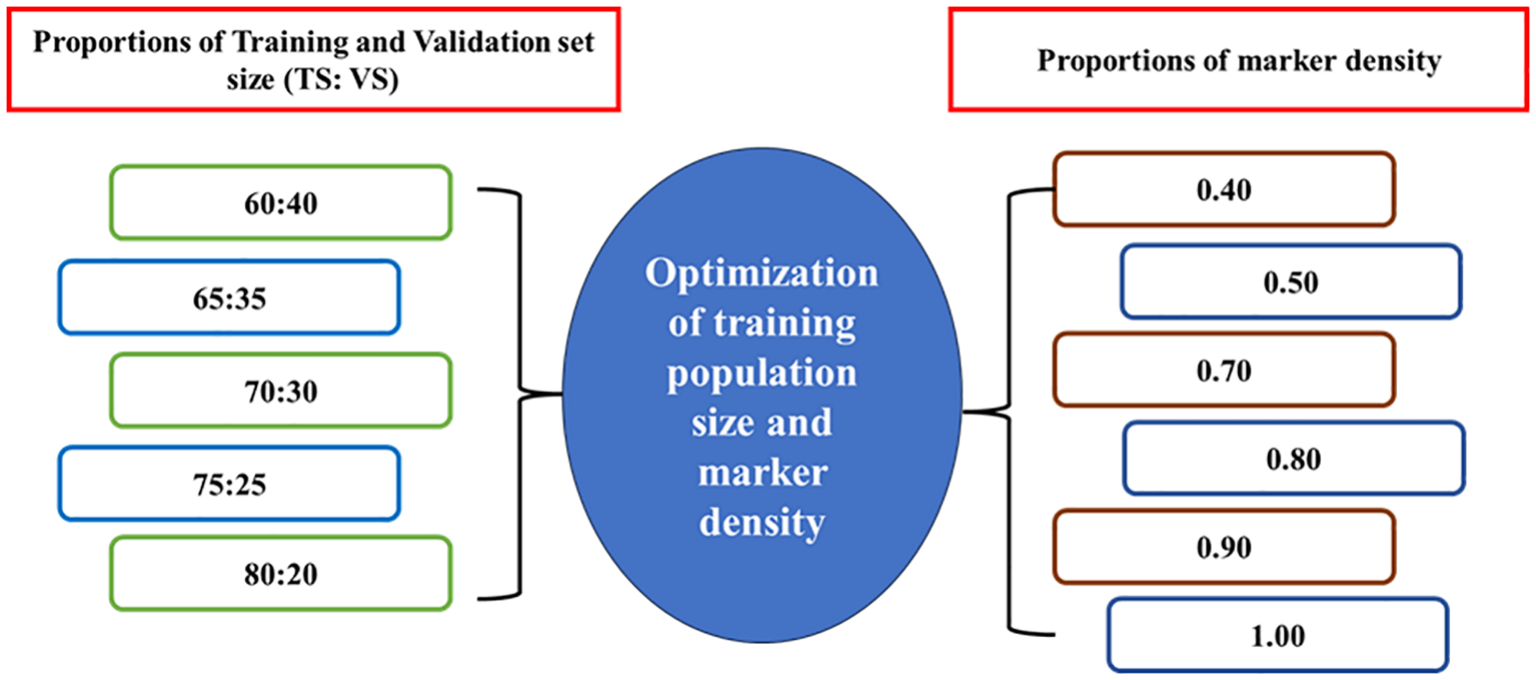
Optimization of training population size and marker density on prediction accuracy.

The effect of marker density on the accuracy of GEBVs was assessed through five-fold cross-validation. Various proportions of training and validation set size are used for optimization keeping marker density constant (100%) as given in **Figure 1**. This procedure was carried out with 1,00,000 iterations such that GEBVs of all individuals of TP were predicted for each tested proportion of training and validation sets.

### 2.5 Estimation of LD decay

Pattern of LD decay in all the three populations was estimated using TASSEL v 5.2.95 (Bradbury et al., 2007) and fitted the LOESS curve using the ggplot2 package (Wickham, 2016) available in R software version 4.4.3. LD decay was estimated by keeping a threshold value of r^2^ value of 0.2 as it is considered biologically meaningful but not due to background noise (Flint-Garcia et al., 2003; Remington et al., 2001).

### 2.6 Independent validation strategy

The marker effects estimated from the prediction models and pooled disease score data from the DHF_1_ and DHF_2_ populations of the cross VL1043 × CM212 using six parametric models were used to predict the FSR disease resistance of individuals of the DHF_2_ population of the cross VL121096 × CM202 (**Supplementary Figure 1**). To further assess the accuracy of the prediction model based on BRR as the estimated prediction accuracy was relatively higher for this model across the populations, 63 random DH lines from all the disease response classes (5 resistant, 34 moderately resistant, 19 moderately susceptible and 5 susceptible) were test crossed with the testers (MAI105 and SKV50) (**Supplementary Figure 2**). The test cross progenies along with the inbreds were evaluated for their disease response. Correspondence between the mean disease score of the test cross progenies with the estimated genomic assisted breeding values of the inbreds was assessed by Pearson’s correlation coefficient.

## 3 Results

### 3.1 Phenotypic variations

#### 3.1.1 Analysis of variance

The FSR response of DH lines (DHF_1_ and DHF_2_) in the individual seasons were subjected to ANOVA (**Tables 2A**, **2B**). The sum of squares due to genotypes was significant in all the seasons in all the DH populations, except in the rainy season of 2019 in DHF_1_ of VL1043 × CM212. Pooled ANOVA across all seasons for all the three DH populations is given in **Table 3**. Non-significance of the mean sum of squares attributable to check with season interactions indicated the absence of GEI (Genotype by Environment Interactions). Thus, average adjusted means across the seasons were considered for calculating pooled mean and it was considered for further analysis.

**Table 2a:** Analysis of variance of mean FSR disease scores of DH lines induced from F_1_ of the cross VL1043 × CM212 in individual seasons.

Source of variation	Mean sum of squares			
	Degrees of freedom	S1	S2	S3
Genotype (ignoring blocks)	337	1.70**	1.40	1.47**
Genotype: Check	1	235.49**	297.01**	303.60**
Genotype: Test	335	0.99**	0.41	0.50*
Genotype: Test vs. Check	1	5.64**	38.25**	22.07**
Blocks (eliminating genotypes)	16	0.35	1.25	0.34
Residuals	16	0.16	1.07	0.20

S1, Winter season of 2018-19; S2, Rainy season of 2019; S3, Winter season of 2019-20.

* and ** indicate significance at 5 and 1 per cent, respectively.

**Table 2b:** Analysis of variance of mean FSR disease scores of DH lines induced from F_2_ of the cross VL1043 × CM212 and VL121096 × CM202 in individual seasons.

Source of variation	Mean sum of squares					
	DHF_2_ of VL1043 × CM212			DHF_2_ of VL121096 × CM202		
	Degrees of freedom	S1	S2	Degrees of freedom	S1	S2
Genotypes (ignoring blocks)	281	1.44**	1.42**	95	2.21**	2.28**
Genotypes: Check	1	190.01**	208.19**	1	132.95**	120.54**
Genotypes: Test	279	0.63**	0.58**	93	0.62*	0.88**
Genotypes: Test vs. Check	1	37.40**	29.77**	1	19.80**	14.33**
Blocks (eliminating Genotypes)	13	0.19	0.10	8	0.21	0.09
Residuals	13	0.08	0.18	8	0.16	0.15

S1, Rainy season of 2019; S2, Winter season of 2019-20

* and ** indicate significance at 5 and 1 per cent, respectively.

**Table 3:** Pooled ANOVA across seasons for the DHF_1_ and DHF_2_ of the cross (VL1043 × CM212) and DHF_2_ of the cross (VL121096 × CM202).

SV	Mean sum of squares					
	Df	DHF_1_ (VL1043 × CM212)	Df	DHF_2_ (VL1043 × CM212)	Df	DHF_2_ (VL121096 × CM202)
Blocks	16	1.52	13	2.93*	8	1.57
Checks	1	58.74***	1	92.75**	1	34.16**
Seasons	2	27.13***	2	1.13	1	0.13
Block × season	32	17.34	26	0.14	8	0.23
Check × Season	2	7.37	2	0.36	1	0.20
Residual	1056	1483.99	879	1.21	204	1.87

Df, Degrees of freedom.

* and ** indicate significance at 5 and 1 per cent, respectively.

#### 3.1.2 Descriptive statistics, genetic variability parameters and comparison of DH lines derived from F_1_ and F_2_ populations

**Figure 2:**
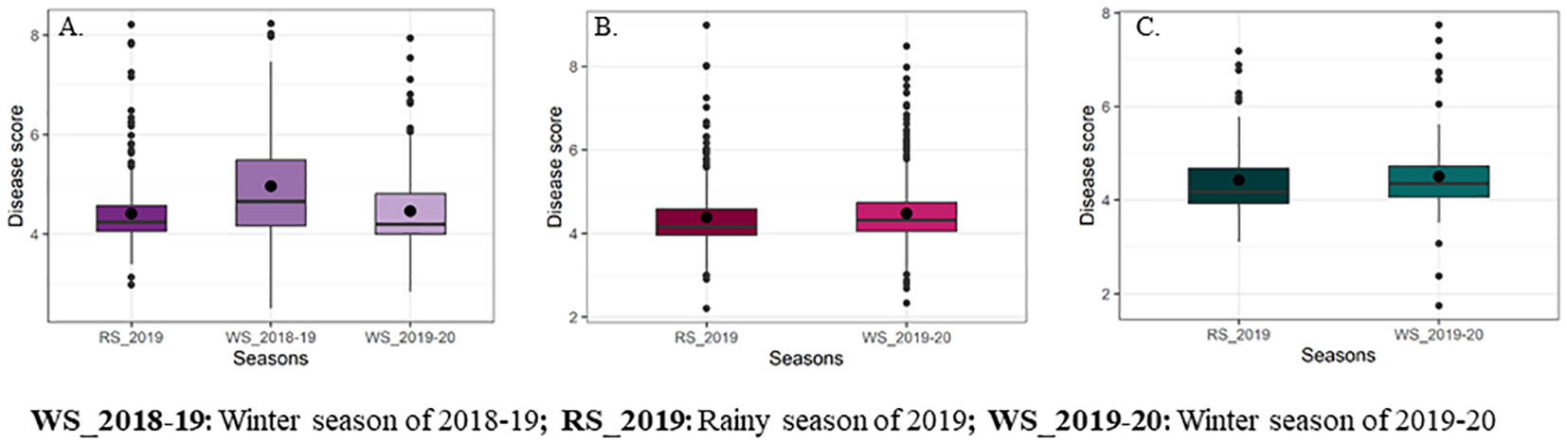
Box whisker plots representing mean disease scores for Fusarium stalk rot reaction of DH populations [**(A)** DH derived from F_1_ of VL1043 × CM212, **(B)** DH derived from F_2_ of VL1043 × CM212 and **(C)** DH derived from F_2_ of VL121096 × CM202].

The descriptive statistics and genetic variability parameters for the response to FSR in DHF_1_ (VL1043 × CM212) and DHF_2_s [(VL1043 × CM212) and (VL121096 × CM202)] are presented in **Table 4** and **Figure 2**.

**Table 4:** Descriptive statistics and estimates of genetic components in maize doubled haploids induced from F_1_ and F_2_ of VL1043 × CM212 cross and F_2_ of VL121096 × CM202 for FSR.

Genetic parameters	DHF_1_ (VL1043 × CM212)			DHF_2_ (VL1043 × CM212)		DHF_2_ (VL121096 × CM202)	
	S1	S2	S3	S2	S3	S2	S3
Mean	4.37	4.43	4.46	4.38	4.52	4.44	4.52
Range	2.84 - 7.03	2.50 - 7.59	2.32 – 8.50	3.15 - 8.75	2.75 - 8.90	2.35 - 7.52	2.10 - 8.69
SR	1.04	1.14	1.38	1.27	1.36	1.16	1.45
CV (%)	8.04	12.90	9.88	6.45	9.17	8.64	8.34
Vg	0.53	0.48	0.30	0.55	0.40	0.46	0.53
PCV (%)	16.99	14.44	15.89	18.17	16.77	17.72	19.85
GCV (%)	15.28	14.13	12.31	16.93	13.92	15.28	18.86
H^2^ (%)	70.0	71.0	69.0	71.0	73.0	74.0	76.0
GAM (%)	34.49	21.56	19.68	32.54	23.83	27.17	35.41

S1, Winter season of 2018-19; S2, Rainy season of 2019; S3, Winter season of 2019-20

SR, Standardized range; CV, Coefficient of variation; PCV, Phenotypic Coefficient of variation; GCV, Genotypic Coefficient of variation; H^2^, Broad sense Heritability; GAM, Genetic Advance as percent mean.

The average standardized range for FSR disease response in DHF_1_ of VL1043 × CM212 across three cropping seasons was 1.18 while that of DHF_2_ of VL1043 × CM212 and VL121096 × CM202 was 1.32. Further, the average genetic variance (Vg) across the seasons in DHF_1_ and DHF_2_ of VL1043 × CM212 were 0.44 and 0.48, respectively. However, DHF_2_ of VL121096 × CM202, the recorded average genetic variance was 0.49.

The estimated genetic parameters *viz*., phenotypic (PCV) and genotypic coefficient of variations (GCV) were moderate in all three DH populations across seasons. The average PCV across seasons in DHF_1_ and DHF_2_ of VL1043 × CM212 were 15.77 and 17.47%, respectively. Whereas, in DHF_2_ of VL121096 × CM202 average PCV was 18.78%. Similarly, the average GCV estimates were 14.04, 15.42 and 16.97% in DHF_1_ and DHF_2_ of VL1043 × CM212 and DHF_2_ of VL121096 × CM202, respectively. Broad sense heritability estimates were high in all three DH populations, with values being 70.0, 72.0 and 75.0% in DHF_1_ and DHF_2_ of VL1043 × CM212 and DHF_2_ of VL121096 × CM202, respectively. Whereas the genetic advance as a per cent mean was moderate (in DHF_1_ of VL1043 × CM212 during the winter season of 2019 - 20) to high across the seasons (**Table 4**).

### 3.2 Prediction accuracy for FSR resistance using five-fold cross validation

Genomic prediction analysis was performed employing BLUP values for fusarium stalk rot disease response. The BLUEs and BLUPs estimates for DH individuals across all three DH populations is given in **Supplementary Tables 3a–c**.

### 3.3 Effect of training population size on prediction accuracy in cross VL1043 × CM212

**Figure 3:**
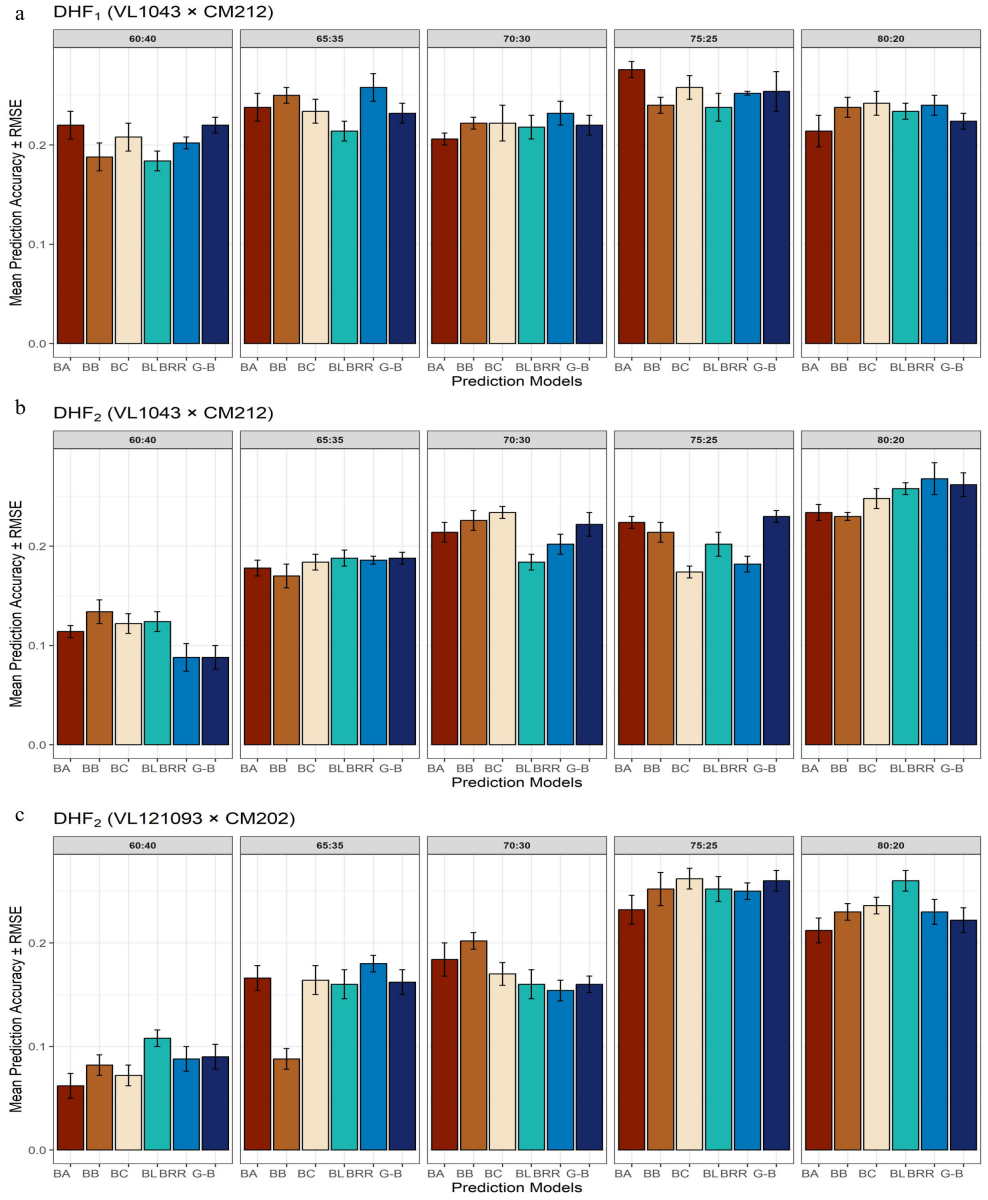
Effect of training population set on estimation of prediction accuracy in three different DH populations [**(a)** DHF1 (VL1043 × CM212), **(b)** DHF2 (VL1043 × CM212), **(c)** DHF2 (VL121096 × CM202].

The DHF_1_ from the cross VL1043 × CM212 consisted of 336 individuals. Prediction accuracies obtained for various proportions of training and validation sets are given in **Figure 3a**. At a 60 TS:40 VS proportion, prediction accuracies estimated from BayesA and GBLUP were similar; however, the estimates were marginally lower in other models. Whereas, at a 65 TS:35 VS proportion, BRR and BayesB models recorded a relatively higher prediction accuracy than in BLASSO, BayesC, and GBLUP. Almost similar magnitudes of prediction accuracies were recorded by all six parametric models at a 70:30 training and validation set proportions. At the 75 TS:25 VS proportion, the highest prediction accuracy was documented by BayesA, followed by BayesC, BRR, GBLUP, BayesB, and BLASSO. The training and validation set proportion of 80:20, recorded comparatively higher prediction accuracy for the BayesB, BayesC and BRR models while the prediction accuracies from the rest models were marginally lower.

The 75:25 proportion of the training and validation set exhibited the highest average prediction accuracy of 0.25 across different TS: VS proportions and the BRR model recorded the highest average prediction accuracy of 0.24.

DHF_2_ from the cross VL1043 × CM212 consisted of 280 individuals. The prediction accuracies recorded for 60:40 proportion of training and validation sets was relatively higher for BayesB followed by BayesC and BLASSO while prediction accuracies from the remaining models were less (**Figure 3b**). The estimated prediction accuracies for the 65:35 proportion were almost similar across all the six prediction models. Whereas, at 70:30 proportions of training and validation sets, the highest prediction accuracy was documented by BayesC and the lowest was by BLASSO. At 75 TS:25 VS proportions, the estimated prediction accuracies were higher in BayesA, BayesB, BayesC, and relatively lower in BLASSO, BRR, and GBLUP.

However, at 80:20 proportions of training and validation sets the average prediction accuracy recorded across all the six models was the highest (0.25). The highest prediction accuracy was recorded by BRR, followed by GBLUP, BLASSO and prediction accuracies in the remaining models was relatively lower. Across varying proportions of TS: VS sets in this cross, GBLUP recorded a higher prediction accuracy of 0.20.

The RMSE error bars around each bar indicate the uncertainty in the prediction. Shorter RMSE error bars indicate consistent and stable performance in prediction across cross-validation folds. Across DHF_1_ and DHF_2_ from the cross VL1043 × CM212, prediction error reduced greatly with increasing training population proportions, suggesting the importance of a larger training population size for effective genomic prediction. The prediction models, BayesB and GBLUP, consistently recorded lower RMSE values, particularly at 75:25 and 80:20 proportions of the training and validation sets. Whereas, the models, BayesA and BLASSO, occasionally documented higher RMSE, indicating greater variability depending upon training population size and population structure (**Figures 3a, b**).

### 3.4 Effect of training population size on prediction accuracy in cross VL121096 × CM202

The DHF_2_ of the cross VL121096 × CM202 consisted of 94 individuals. Prediction accuracies estimated for 60:40 proportion across six different parametric models, were relatively higher and lower in BLASSO and BayesA, respectively. The higher magnitude of prediction accuracy was documented by BLASSO, BayesA, BayesC, BRR and GBLUP while lower magnitude of prediction accuracy was recorded by BayesB for the proportion of 65 TS:35 VS. For the training and validation set proportion of 70:30, the highest prediction accuracy was recorded by BayesA, and the lowest by BRR. For the training and validation set proportions of 75:25, the estimated prediction accuracies were almost similar across all the six models. At 80 TS: 20 VS proportion, the highest prediction accuracy was recorded by BLASSO and the lowest by BayesA (**Figure 3c**). The highest average prediction accuracy was recorded by the prediction model BLASSO (0.19) and the 75:25 proportion of training and validation set (0.25).

**Figure 4:**
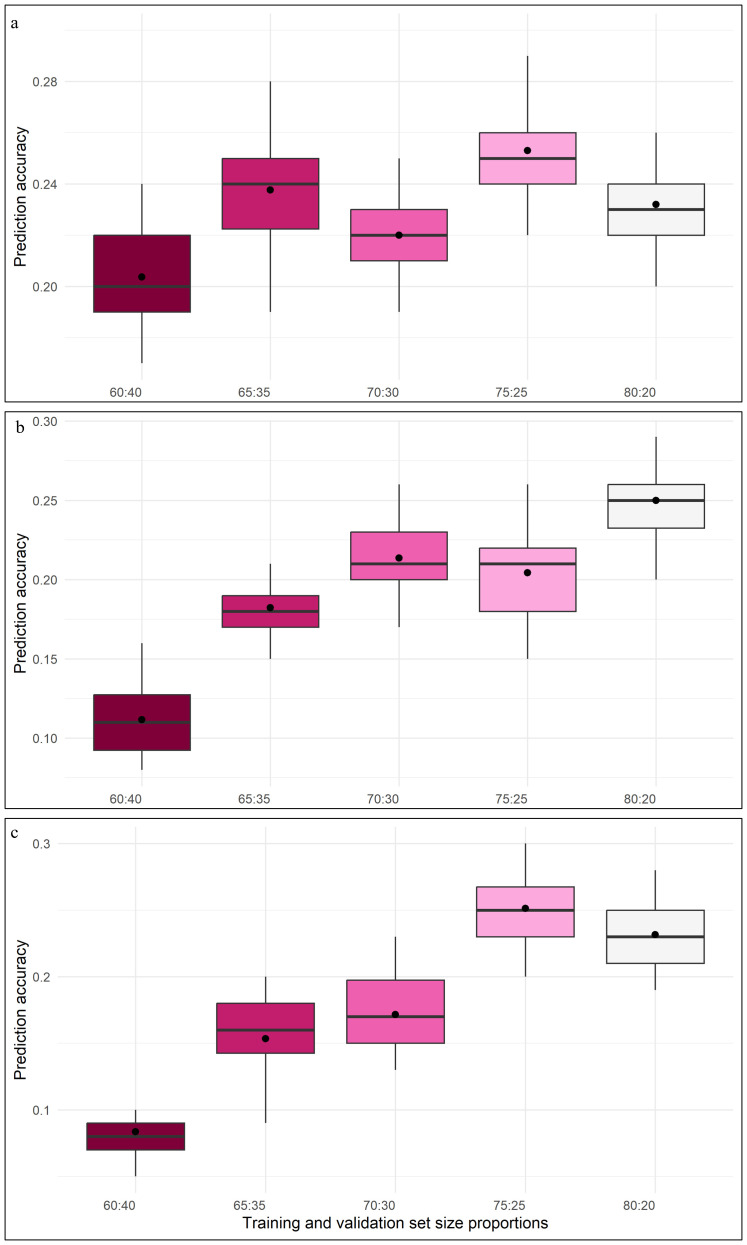
Box plot depicting the variation of prediction abilities across different training and validation set proportions **(a)** DHF1 (VL1043 × CM212), **(b)** DHF2 (VL1043 × CM212), **(c)** DHF2 (VL121096 × CM202).

Among all the considered models, GBLUP and BayesB displayed relatively higher prediction accuracy, coupled with lower RMSE, especially at 75:25 and 80:20 proportions of training and validation sets. Whereas, for 60:40 and 65:35 proportions of training and validation sets, most of the models recorded noticeably larger prediction errors. DHF_2_ from VL121096 × CM202 seemed to respond well to an increase in training population size, which is evident through sharp improvement in prediction accuracy and a reduction in the magnitude of prediction error. The variation in prediction accuracy estimated across five folds for different proportions of training and validation sets is given in the box plots (**Figure 4**).

### 3.5 Effect of marker density on the prediction accuracy

**Figure 5:**
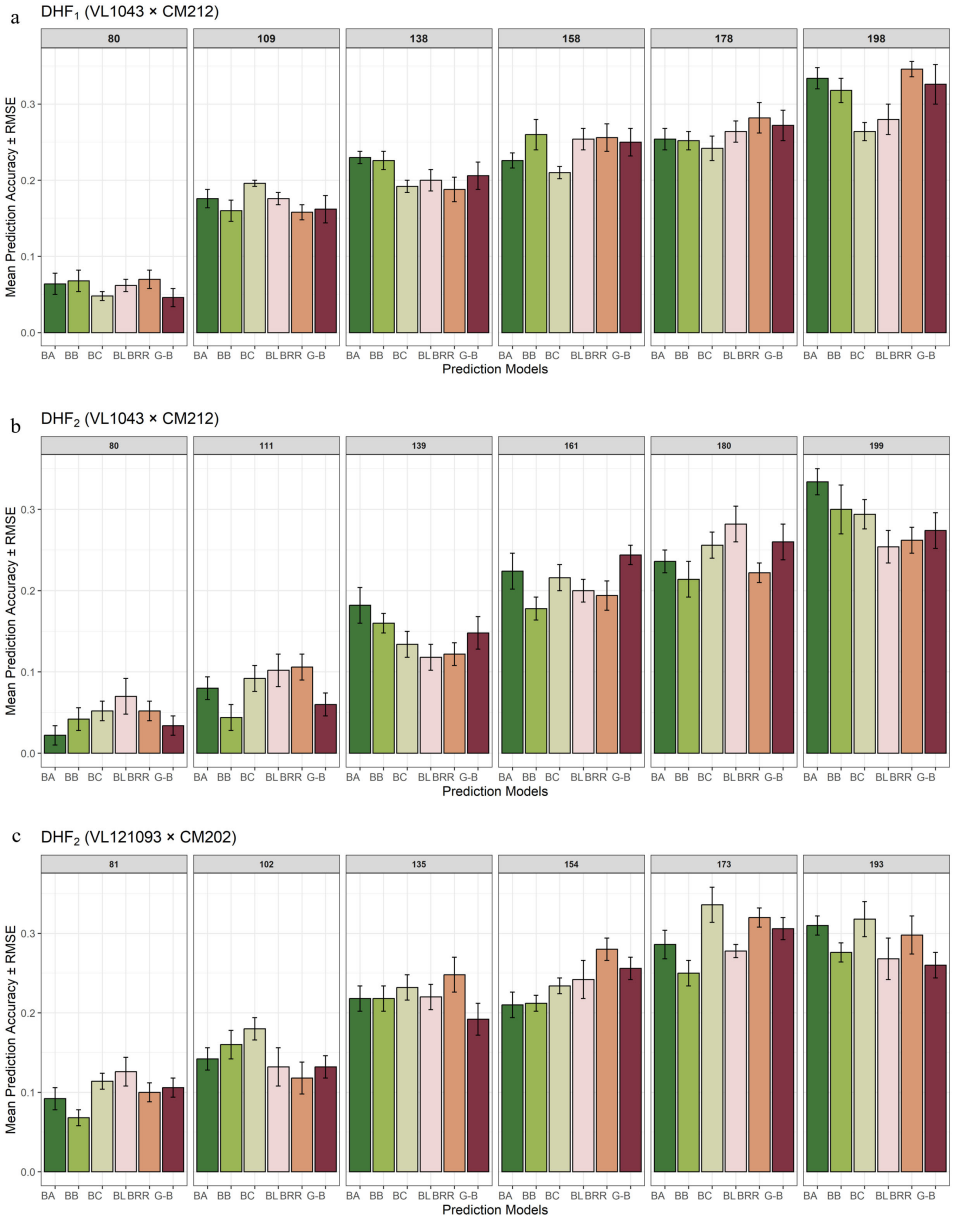
Effect of marker density on the prediction accuracy in three DH populations [**(a)** DHF_1_ of VL1043 × CM212, **(b)** DHF_2_ of VL1043 × CM212 and **(c)** DHF_2_ of VL121096 × CM202.

The estimated prediction accuracies in six different parametric models using five-fold cross validation are given in **Figure 5**. The average prediction accuracies across six different parametric models *viz.*, GBLUP, BayesA, BayesB, BayesC, BLASSO and BRR using five-fold cross-validation in DHF_1_ of VL1043 × CM212 displayed an increasing trend from 0.06 to 0.31 with an increase in marker density from 80 (40%) to 198 (100%) (**Figure 5a**).

A similar pattern of the increasing trend in prediction accuracy with an increase in marker density was observed for DHF_2_ of the cross VL1043 × CM212 [0.04 (41%) to 0.28 (100%)] (**Figure 5b**) and 0.10 to 0.30 for the DHF_2_ of the cross VL121096 × CM202 for an increase in marker density from 81 (42%) to 173 (89.63%) (**Figure 5c**). Further, no significant increase in the prediction accuracy was recorded when the 100% marker density was employed to predict the prediction accuracy in DHF_2_ of VL121096 × CM202.

Higher prediction accuracy was recorded when 100% of the marker density was employed in DHF_1_ and DHF_2_ of VL1043 × CM212. However, in the DHF_2_ of cross VL121096 × CM202, the highest prediction accuracy was recorded for 89.63% (173 markers) marker density and no further improvement in prediction accuracy was noted.

**Figure 6:**
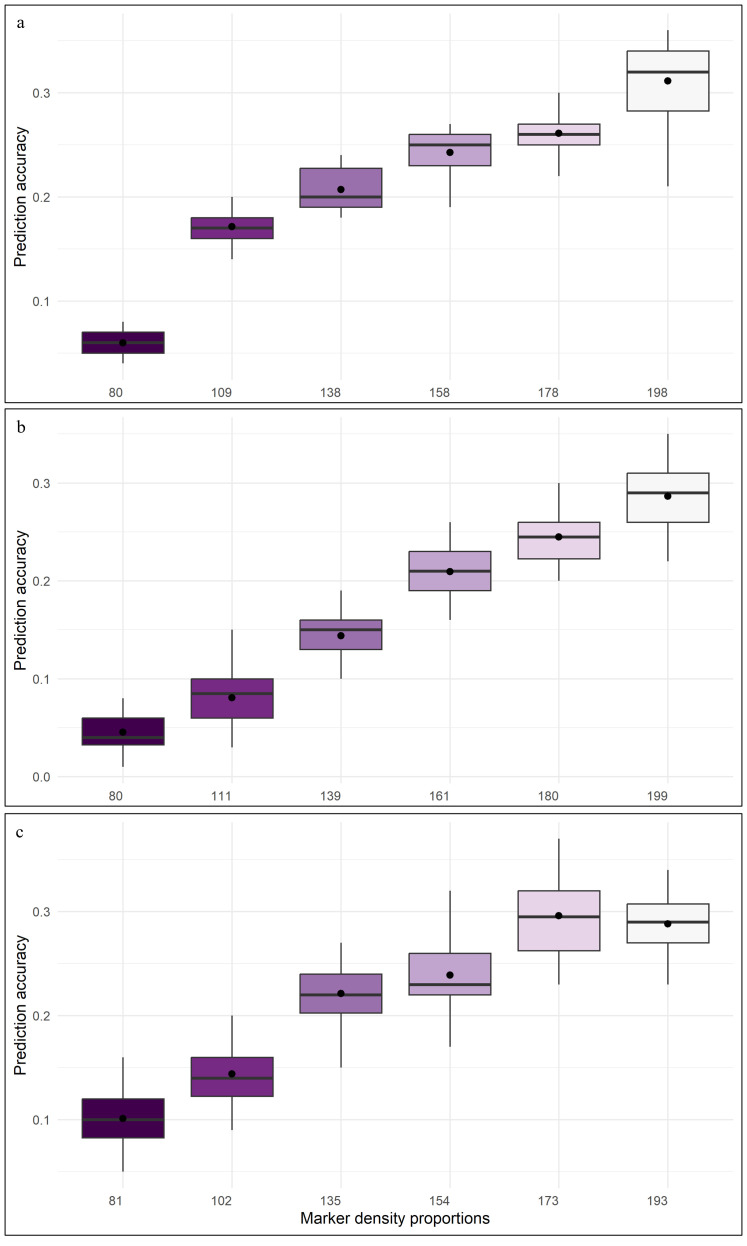
Box plot depicting the variation of prediction abilities across different marker density proportions **(a)** DHF1 (VL1043 × CM212), **(b)** DHF2 (VL1043 × CM212), **(c)** DHF2 (VL121096 × CM202).

Estimated prediction errors across models and marker densities revealed that at lower marker densities, most of the models exhibited higher prediction error (RMSE), indicating greater prediction variability. It was observed that with an increase in marker density, the RMSE decreased substantially, particularly in models like GBLUP and BayesB, which consistently produced a stable prediction value with minimal errors. Reduction in RMSE with an increase in marker density underlined the importance of using adequate genome coverage markers for minimizing the prediction error in genomic prediction studies. The box plot showing variation in prediction accuracy across five-fold for various tested marker densities is given in **Figure 6**.

### 3.6 LD decay and its effect on marker density

The LD decay patterns across all the three populations were estimated at a threshold r^2^ value of 0.2 (**Supplementary Figure 3**). LD decay distance of 7, 13 and 31cM were obtained for DHF_1_ from VL1043 × CM212, DHF_2_ from VL1043 × CM212 and VL121096 × CM202, respectively.

Optimum number of markers to be used for effective capturing of all the genetic variation was estimated using the information on LD decay (dividing the genetic map length by the LD decay value). The results found that approximately 286, 154 and 65 SNPs were sufficient for effective estimation of prediction accuracy. It was evident from the estimated prediction accuracies across different proportions of marker densities tested, the highest prediction accuracy was recorded for the marker density of 85–100 per cent across all the DH populations.

### 3.7 Independent validation of the GS model

**Figure 7:**
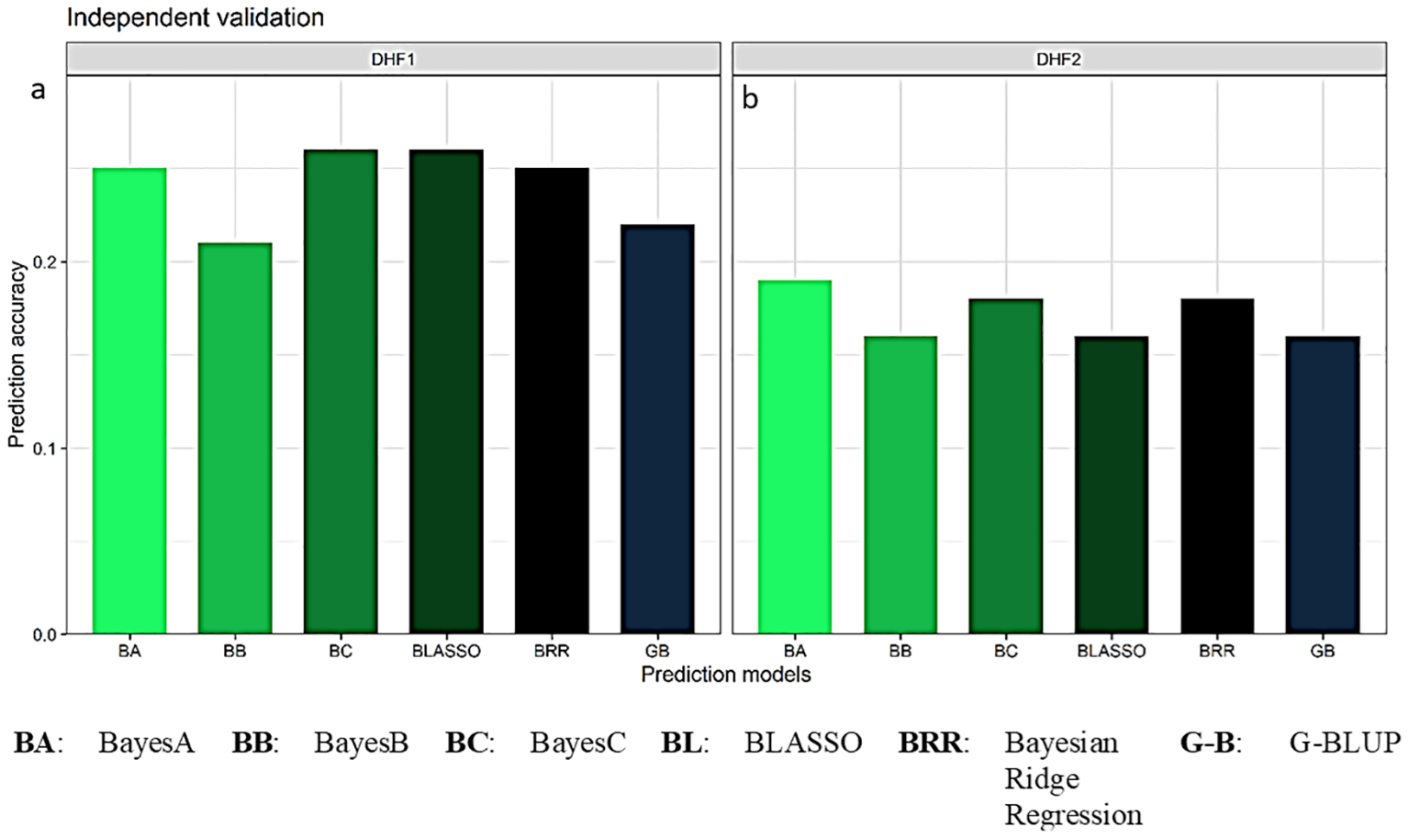
Prediction accuracy for independent validation of DHF_2_ of VL121096 × CM202 using **(a)** DHF_1_ of VL1043 × CM212 and **(b)** DHF_2_ of VL1043 × CM212 as training sets.

The prediction accuracies estimated for FSR resistance in independent validation when the DHF_2_ of the cross VL121096 × CM202 was used as a validation set and the DHF_1_ and DHF_2_ of the cross VL1043 × CM212 were used as training sets. The average prediction accuracy estimate of 0.24 was documented in the independent validation of DHF_2_ of the cross VL121096 × CM202 using DHF_1_ of the cross VL1043 × CM212 as the training set. The prediction accuracies in five-fold cross validation using six different parametric models were 0.22, 0.25, 0.21, 0.26, 0.26 and 0.25 in GBLUP, BayesA, BayesB, BayesC, BLASSO and BRR, respectively (**Figure 7a**). The average prediction accuracy of 0.17 was recorded when DHF_2_ of the cross VL1043 × CM212 was used to train the model. Prediction accuracies estimated in five-fold cross validation across six parametric models *viz.*, GBLUP, BayesA, BayesB, BayesC, BLASSO and BRR were 0.16, 0.19, 0.16, 0.18, 0.16 and 0.18 (**Figure 7b**).

### 3.8 Evaluation of test cross progenies

A total of 63 DH lines from all disease response class were chosen randomly and crossed with two testers namely MAI105 and SKV50 to derive the test cross progenies and their disease response was assessed phenotypically.

**Figure 8:**
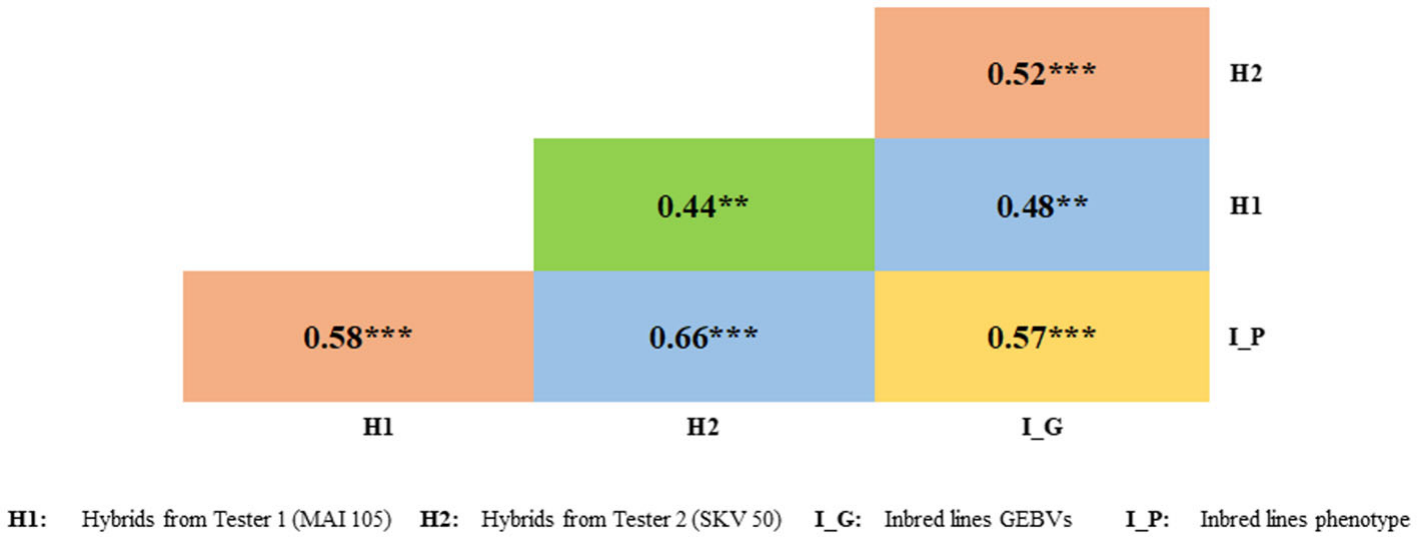
Correlation coefficients between phenotypic values of testcross progenies with selected inbred line's phenotypic values and GEBVs. ** and *** indicate significance at 1 and 0.1 per cent, respectively.

A significant positive correlation was documented between GEBV’s with the phenotype (0.57) of the selected DH lines, GEBV’s with the test cross progenies derived by crossing with the testers MAI105 (0.48) and SKV50 (0.52) was documented. The estimated Pearson’s correlation coefficient between the disease expression of selected lines with the test cross progenies derived by crossing the selected lines with two testers MAI105 and SKV50 were 0.58 and 0.66, respectively (**Figure 8**).

## 4 Discussion

Doubled haploid (DH) technology has emerged as an efficient strategy to shorten breeding cycles significantly and increase genetic gain (Chaikam et al., 2019). The application of genomic prediction in conjunction with DH technology is known to accelerate the pace of achieving targeted genetic gain (Fu et al., 2022). Identifying and utilization of the lines displaying resistance to an important disease like Fusarium stalk rot in maize is very crucial as this disease is prevalent in most maize-growing areas.

### 4.1 Impact of genetic variations in DH lines derived from F_1_ and F_2_ populations

The significance of the mean sum of squares due to genotypes in the three DH populations indicated the presence of a substantial amount of genetic variability in the material considered for the study. Further, pooled ANOVA across seasons in the three DH populations indicated the non-significance of check × season interactions indicating the absence of genotype by environment interactions. Thus, average adjusted disease scores across seasons were considered for calculating the pooled mean which was used for further genomic selection analyses.

The mean and standardized range of FSR disease scores of DHF_2_s were greater than the DHF_1_ in both crosses. The range was relatively wider in DHF_2_s compared to DHF_1_s indicating the presence of higher variability among DHF_2_s than DHF_1_. These results were expected due to an additional round of recombination in F_2_ which contributed to an increase in the genetic variability in DHF_2_s (Chase, 1969; Chalyk, 1994; Rotarenco et al., 2007; Geiger et al., 2013; Sleper and Bernardo, 2016; Couto et al., 2019).

Estimating genetic variance is important in predicting the response to selection, understanding the gene action of quantitative traits and for effective planning of the breeding procedure (Choo, 1980). The genetic variation between DH lines gives the estimates of additive components of FSR resistance. Within the DHF_1_ and DHF_2_s of the cross VL1043 × CM212 genetic variance was higher in DHF_2_ than in DHF_1_. However, the genetic variance (V_g_) in DHF_2_ of VL121096 × CM202 cross, was higher than both DHF_1_ and DHF_2_ s of the cross VL1043× CM212. The differences in genotypic variances between DHF_1_ and DHF_2_ could be attributed to an additional round of recombination. Further, linkage causes the Vg to differ between DHF_1_ and DHF_2_ lines. Coupling phase linkage leads to larger Vg among DHF_1_ lines than among DHF_2_ lines, and it is apparent by a decrease in the proportion of extreme types, a situation characteristic of the breaking of coupling phase linkages. Whereas, the repulsion linkage leads to a larger Vg among DHF_2_ lines than among DHF_1_ lines regardless of the type of gene action and the hidden genetic variance that is released upon the disruption of repulsion linkages, and this reflects higher proportions of extreme genotypes in the DHF_2_s at both ends of phenotypic distribution in that cross. A relatively higher magnitude of Vg in the DHF_2_s than in DHF_1_ indicates the presence of repulsion linkages in the genetic control of FSR resistance (Weir et al., 1980; Sleper and Bernardo, 2016). Generally, the F_2_ generation is the superior segregating population to initiate DH production. Extrapolation of these results to other elite line crosses should be cautioned since conclusions drawn are specific to the germplasm used. Future studies using other elite inbred lines should provide evidence for trends regarding the superior type of segregating population employed in DH production.

The genotypic coefficient of variation and phenotypic coefficient of variation are standardized estimates of variability at genotypic and phenotypic levels, respectively. Both GCV and PCV estimates owing to their unit independence, facilitate the better comparison of variability. The estimates of PCV and GCV were lower in DHF_1_ compared to DHF_2_s. The higher variation of DHF_2_ compared to DHF_1_ was probably due to the additional round of recombination later than the prior (Snape and Simpson, 1981). Further, the close correspondence between GCV and PCV indicated the lesser influence of the environment on the expression of FSR disease reaction, and selection based on the phenotype performance would be effective (Chacko et al., 2023). All three populations recorded a higher estimated broad sense heritability coupled with moderate to higher genetic advance as per cent mean implying reward to selection practiced.

### 4.2 Estimation of genomic estimated breeding values and prediction accuracies

#### 4.2.1 Effect of TP size on the accuracy of predicted GEBVs

General consensus does not exist in the literature regarding the optimum size of the TP to achieve high accuracy of predicted GEBVs. However, acceptable GEBV prediction accuracy was achieved in maize bi-parental populations using as few as 60 (Schaeffer, 2006) and 84 (Riedelsheimer et al., 2013) individuals. Thus, the use of a DH population consisting of 336, 280 individuals in F_1_ and F_2_ induced DHs of the cross VL1043 × CM212 and 94 individuals in F_2_ induced DHs of the cross VL121096 × CM202 in the present study for predicting and validating GEBVs for FSR resistance is justified.

Further, DH populations are frequently used for selection in predominantly cross-pollinating crops like maize. Assessing the accuracy of predicted GEBVs in such populations will directly affect the efficiency of maize breeding. Population structure is of no concern if DH populations are used for predicting and validating GEBVs since all the individuals are true to type and completely homozygous (Lorenzana and Bernardo, 2009). Hence, all three crosses were used as TP, to understand the effect of TP size on the accuracy of predicted GEBVs.

In the present study, the TP was progressively increased by dividing the TP into a training and validation set in different proportions, such as 60:40, 65:35, 70:30, 75:25 and 80:20 in favour of TS and VS, respectively, keeping the marker density at 100% for optimizing the composition of training population size.

In DHF_1_ and DHF_2_ from the crosses VL1043 × CM212 and VL121096 × CM202, the highest prediction accuracy was obtained for the training and validation set proportion of 75:25. Whereas, in DHF_2_ of the cross VL1043 × CM212 the highest prediction accuracy was recorded for 80:20 proportion of training and validation sets. Thus, the highest prediction accuracy was recorded when nearly 75 - 80% of the individuals were used for training the models in both the populations in five-fold cross validation. Increasing trend of prediction accuracy was observed for fusarium stalk rot resistance with increase in proportion of training population from 20 to 80% (Song et al., 2024). Larger sizes of training sets reduce the bias and reduce the variance of marker effect estimates, thereby increasing the prediction accuracy. A small training set size leads to overfitting, wherein the marker effects are fitted to noise rather than true genetic signals, whereas the use of larger training sets provides a better signal-to-noise ratio and captures wider genetic variability, thereby improving the model’s generalizability to new individuals (de los Campos et al., 2013). Further, optimization of the training population size by Islam et al. (2020) in cotton revealed that prediction accuracy was highest for the 90:10 proportion of training and validation sets. Further, Zhang et al. (2017) studied the effect of marker density and training set size on prediction accuracy for three different agronomic traits like plant height, days to anthesis, and grain yield under well-watered and water stressed conditions and also observed an increase in prediction accuracy with an increase in training set size and marker density. A similar study by Fan et al. (2024) on flowering time related traits in an association mapping panel of 379 DH lines showed the highest prediction accuracy when 70% of the population was used for model training. The optimum size of the training population needed for training the model depends upon the genetic architecture of the trait (Gilmour, 2007).

GBLUP and BayesB models outperform other prediction models in terms of higher prediction accuracy and lower RMSE, while dealing with structured populations. High relatedness among individuals of a DH population enhances the GBLUP’s ability to capture additive genetic variance through the genomic relationship matrix effectively (Habier et al., 2007). Whereas, BayesB model assumes only a smaller proportion of markers have large effects while rest of the markers have zero effect on target trait. This model conducts variable selection which aids in noise reduction from non-informative markers, thereby enhancing the model’s performance (Meuwissen et al., 2001).

#### 4.2.2 Effect of marker density on the accuracy of predicted GEBVs

It is reported that marker density impacts GEBV’s prediction accuracy in genomic selection (Bernardo and Yu, 2007; Nakaya and Isobe, 2012; Crossa et al., 2014). Higher prediction accuracies were recorded for 100% marker density in DHF_1_ and DHF_2_ of the cross VL1043 × CM212. Whereas, in DHF_2_ of a cross VL121096 × CM202, higher prediction accuracy was documented for 89.63% (173 markers) marker density. The increasing trend of prediction accuracy estimation with increasing marker density could be due to the fact that with more markers, the probability of identifying the causative loci influencing the trait will increase. Further, dense marker panel lead to accurate estimation of relatedness, improves the efficiency of GEBV estimation and reduces the bias in estimation of marker effects (Daetwyler et al., 2008).

In maize (Vivek et al., 2017) and barley (Lorenzana and Bernardo, 2009) it was demonstrated that GEBV prediction accuracy increased with increasing the number of markers in DH populations. However, the increase was large only at low marker densities. For example, the accuracy of predicted GEBVs for grain protein content increased significantly with the number of markers from 64 to 128; however, the accuracy did not change from 128 to 223 markers. Further, a study by Cao et al. (2021) showed that higher marker density slightly improved prediction accuracy for tar spot complex disease in maize; however, the increase was not substantial. This suggests that a moderate number of well-distributed markers may be sufficient for effective genomic selection. However, as reported by several researchers in different crops, the possibility of increasing the accuracy needs to be explored by using large sizes of the TP. The marker density threshold might be determined by the extent of linkage disequilibrium (LD) between the markers and the QTL in the genome (Wang et al., 2017). Strong LD between marker alleles and causal QTL in DH populations allow localization of QTL to large intervals (10–20 cM) in the genome. Each marker allele is potentially in LD, with at least one causal QTL controlling the target trait (Morgante et al., 2018). Theoretically, the extent of LD in a population is a function of effective population size (Syed, 1971; Wientjes et al., 2013). At a low effective population size, the number of independent genome segments is expected to be small; hence, fewer markers are sufficient to mark all the genome segments (Goddard and Meuwissen, 2010; Poland et al., 2012). The magnitude of prediction accuracy obtained for FSR resistance in the present study was comparable to that reported in the literature for northern corn leaf blight resistance (0.11 – 0.29) in a cross validation (Technow et al., 2013).

In the present study, the population size of the F_2_-derived DHs of VL121096 × CM202 was relatively small. Since DHs are full-sib progenies, it is possible that large segments share similar genome sequences such that they share marker alleles identical by descent (Poland et al., 2012), leading to marker redundancy as the number of markers increases (Peixoto et al., 2016). Several studies have demonstrated high GEBV prediction accuracy for many traits like northern corn leaf blight resistance (Lohithaswa et al., 2024), grain yield, plant height, and flowering time (Spindel et al., 2015) using fewer markers. However, much research is required to optimize the number of markers to realize maximum prediction accuracy and genetic gain using GS in different training populations, their composition and size, and prediction models.

Though the estimated heritabilities for FSR disease response were high, prediction accuracies were low to moderate that could be due to strong relatedness among the individuals (Liu et al., 2017), smaller training population sizes (Crossa et al., 2025; Vieira et al., 2025) and complexity of genetic architecture (Crossa et al., 2017; Zhang et al., 2015). The trait with high heritability might be influenced by rare alleles or alleles with non- additive genetic effects, which are not well captured by the models that assume additive genetic effects such as GBLUP (Jiang et al., 2018). Along with that, relatedness between the training and breeding population, marker density and genome coverage, genetic diversity within the training population, linkage disequilibrium, choice of prediction models, inclusion of genotype by environment interactions and type of marker used also influence the prediction accuracy (Crossa et al., 2017, 2014). Further, the training population was derived by crossing only two complementary parents, genetic variability for the target trait may not be effectively captured. It is noted that diversifying the training population will increase the robustness of GEBVs prediction thereby increasing the prediction accuracy in genomic selection studies (Burstin et al., 2015). Lan et al. (2020) found that, even for the traits with high heritability, the accuracy of prediction depends mainly on whether the marker set contains sufficient QTLs to contribute to the total variation of the phenotypes, or whether all the related QTLs have been identified from the marker set. Lozada et al. (2019), observed that the low prediction accuracy was recorded even if the trait has recorded high heritability when the markers used might not have efficiently captured the LD between markers and the QTLs.

Predictive accuracy and reliability of genomic selection models are assessed by cross validation. Further, cross validation also ensures that the model is not overfitting to the training data and it can be generalized to new set of genotypes. Efficiency of different statistical models and machine learning approaches can be assessed through cross validation. Along with this, size and composition of training population can be optimized through cross validation (Friedman et al., 2001). Five-fold cross validation was employed to compare the efficiency of model performance under different marker density and training population proportions. As the number of individuals in the training population increases (less folds) it reduces the variance at each fold. However, as the number of folds increases, the variance of whole cross-validation estimate reduces. However, among various k values in the k fold cross validation, the five-fold and 10-fold cross validation schemes are found to be more reasonable for estimating the marginal predictive errors (Schrauf et al., 2021).

Prediction error RMSE displayed a declining trend with an increase in marker density across all three DH populations, highlighting the critical role of marker density on prediction accuracy. At the lower marker densities, the prediction values were less stable, as indicated by the larger RMSE values, likely due to the insufficient capture of underlying genetic variance. As the marker density increased above 70 per cent, RMSE estimates decreased significantly in GBLUP and BayesB, indicating improved stability and predictive ability with an increase in marker density. The GBLUP model, which exploits the genomic relationship matrix to model additive effects, is generally effective when the population structure is more evident (Habier et al., 2007). BayesB model’s sparse variable selection strategy can identify major effect loci and ignore the non-informative markers, resulting in enhanced robustness even under variable genomic marker densities (Meuwissen et al., 2001). These findings are consistent with earlier studies showing that both high marker coverage and appropriate model choice are essential for achieving low prediction error and high accuracy in genomic selection (Zhang et al., 2017).

Further, the average prediction accuracies obtained after assessing the effect of training population size and marker density differed greatly. The average prediction accuracy after optimizing the marker density was relatively higher than that obtained for the training population proportion. In a crop like maize with a high LD decay, the margin of increase in prediction accuracy is higher for marker density than the proportion of training population used (Bellon et al., 2018; Moghaddam and Morrel, 2018; Crossa et al., 2017). Further, for the polygenically controlled traits, especially in a population with a low LD, increasing marker density has a positive effect on prediction accuracy estimation (Habier et al., 2007).

### 4.3 LD decay and marker density

The effect of marker density on the accuracy of GS prediction is the most researched element, and it is agreed that a higher number of markers typically produces higher accuracy up to a plateau (Meuwissen et al., 2001; Habier et al., 2007; Daetwyler et al., 2008; Zhang et al., 2015; Krishnappa et al., 2021). In the present study, marker density employed was comparatively low, and hence, linkage disequilibrium (LD) was considered to estimate the optimum marker density, as it is known to play a crucial role in determining the optimum marker density needed for genomic selection. The relationship between the LD and marker density directly impacts the accuracy of GEBVs and the efficiency of the genomic prediction models (Liu et al., 2015). To address the effect of LD decay on marker density, LD decay pattern of all the three DH populations was carried out. According to the estimated LD decay value, the marker density employed was sufficient for DHF_2_s from the crosses VL1043× CM212 and VL121096 × CM202. The optimum number of markers for effective estimation of prediction accuracy was computed by dividing the average genetic map length of these populations with their respective LD decay values (Kanaka et al., 2023). The genetic map length of DHF_2_ of VL1043 × CM212 was 2156.36 cM and that of VL121096 × CM202 was 2100.18 cM (Showkath Babu et al., 2024). Hence, the average genetic map length of 2000 cM was considered to calculate the optimum number of markers. It was evident that the estimated prediction accuracy of the DH populations (DHF_2_s of VL1043 × CM212 and VL121096 × CM202) was the highest for the marker density of 85–100 per cent indicating that the number of markers used in the present study was sufficient. Whereas, the LD decay value of DHF_1_ from VL1043 × CM212 was very low, indicating the need for further increasing the marker density. However, the prediction accuracy of this cross was comparable with the prediction accuracy estimated for other two populations.

### 4.4 Comparison of models’ performance

Across different marker densities, the Bayesian alphabets (BayesC and BayesA) and Bayesian ridge regression (BRR) gave comparatively higher prediction accuracies. Comparatively, better performance of Bayesian models could be due to the basic assumptions these models hold. Bayesian models effectively distinguish between the truly important markers and background noise (Gianola and de los Campos, 2008). The GBLUP model, assumes that all markers have effect on trait variability, whereas the Bayesian alphabets assume only a limited number of markers have effect on trait variation. Common variance for all the markers was considered by GBLUP, BayesC and BRR models however, other Bayesian models namely BayesA, BayesB and BLASSO assume specific variances for marker effects (Meher et al., 2022). The comparative effectiveness of the genomic prediction models used is largely influenced by the trait architecture as the models differ in assumptions about the distribution of marker effects (Perez-Rodriguez et al., 2012). It is proved that GBLUP performs well for traits governed by many QTLs each with small effects. On the other hand, the Bayesian alphabets perform well for traits governed by few QTLs each of them having major effect on genetic variability. Meher et al. (2022) proved that GBLUP model was the least biased in prediction accuracy estimation compared to various BLUP and Bayesian model variants.

### 4.5 Independent validation of calibrated GS model

The estimated prediction accuracy was highest in the independent validation when DHF_1_ of the VL1043 × CM212 was used as a training set (0.24). The prediction accuracy of 0.17 when DHF_2_ of the VL1043 × CM212 was used as a training set. These results are only indicative as they are based on fewer individuals in TP and markers and five-fold cross-validation. Dependable results could be obtained based on independent validation in a large number of cross-populations (Osorio et al., 2021).

### 4.6 Evaluation of test cross progenies

Assessing the test cross progenies’ performance offers valuable insights into the translation of genetic predictions into phenotypic expressions, hence providing real-time validation of the practical applicability of genomic selection models (Crossa et al., 2014). Furthermore, for breeding programs to be successful, it is imperative to consider the field performance of the lines selected based on GEBVs.

A significant positive correlation of the GEBV’s with the phenotype of the selected DH lines, and test cross progenies derived by crossing with the testers MAI105 and SKV50 indicated the effectiveness of genomic selection model in identifying the potential lines with resistance to FSR disease. Correlation coefficient can be used as a measure to assess efficiency and robustness of the selection model (Heslot et al., 2012; Schopp et al., 2017). However, in the small sample size random effects can influence the observed correlation leading to over or underestimation of prediction accuracy (Daetwyler et al., 2008).

## 5 Conclusion

The current investigation demonstrated the application and feasibility of genomic selection for genetic improvement in maize for fusarium stalk rot resistance. The training population size and marker density were optimized by testing different proportions of training and validation sets and different marker densities. The estimated descriptive statistics and genetic variability parameters were higher in DHF_2_s than in DHF_1_ populations. Higher prediction accuracy was recorded for 75:25 proportions of training and validation sets and 80 - 100% marker density. Further, independent validation was performed to assess the robustness of the developed models. We showed that it could be possible to get good prediction accuracies with the optimum population size and marker density, instead of the larger population. Further, the test cross hybrids generated using the DH lines selected from different disease response classes displayed a higher correlation coefficient with the phenotypic response and GEBVs of selected lines.

## Data Availability

The datasets presented in this study can be found in online repositories. The names of the repository/repositories and accession number(s) can be found in the article/**Supplementary Material**.
