# Supplementary figures and images for: Assessment of the potential for genomic selection to improve resistance to fusarium stalk rot in maize
Source: Front Plant Sci. 2025 Sep 23;16:1631408. doi: 10.3389/fpls.2025.1631408 (PMC12500717; doi:10.3389/fpls.2025.1631408)

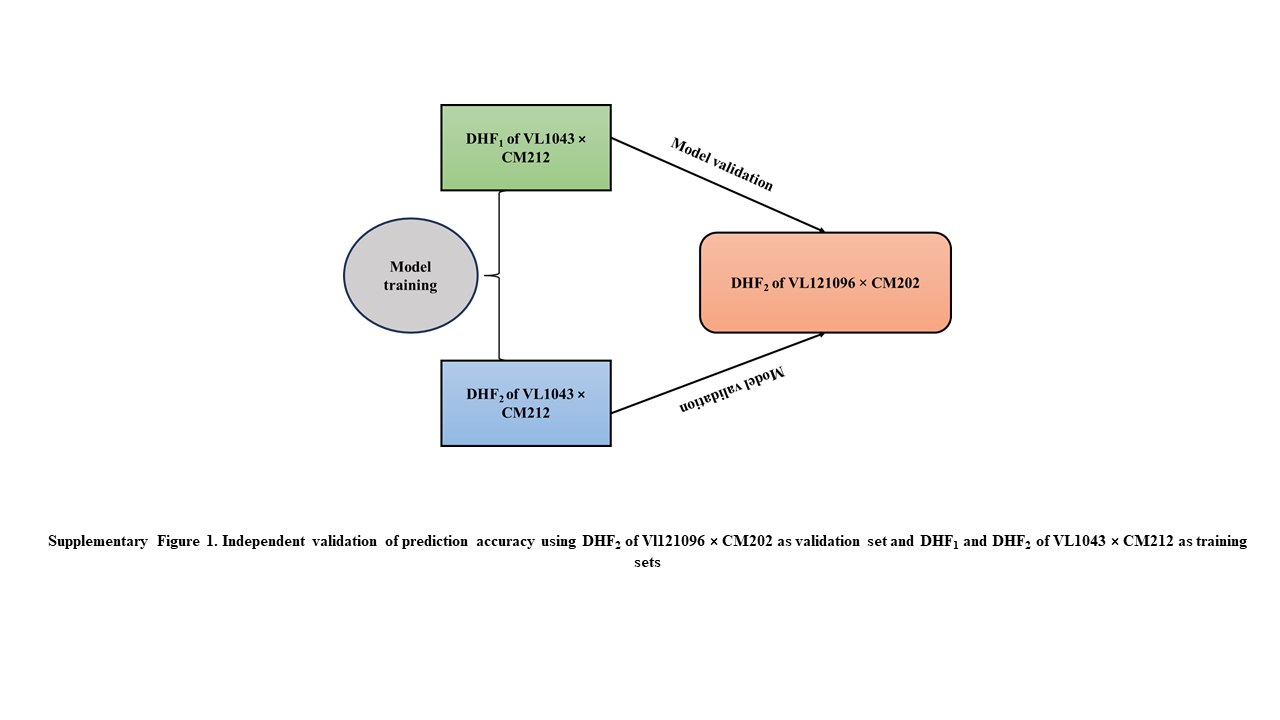

Supplement: Supplementary file 4 [file Image1.tif]

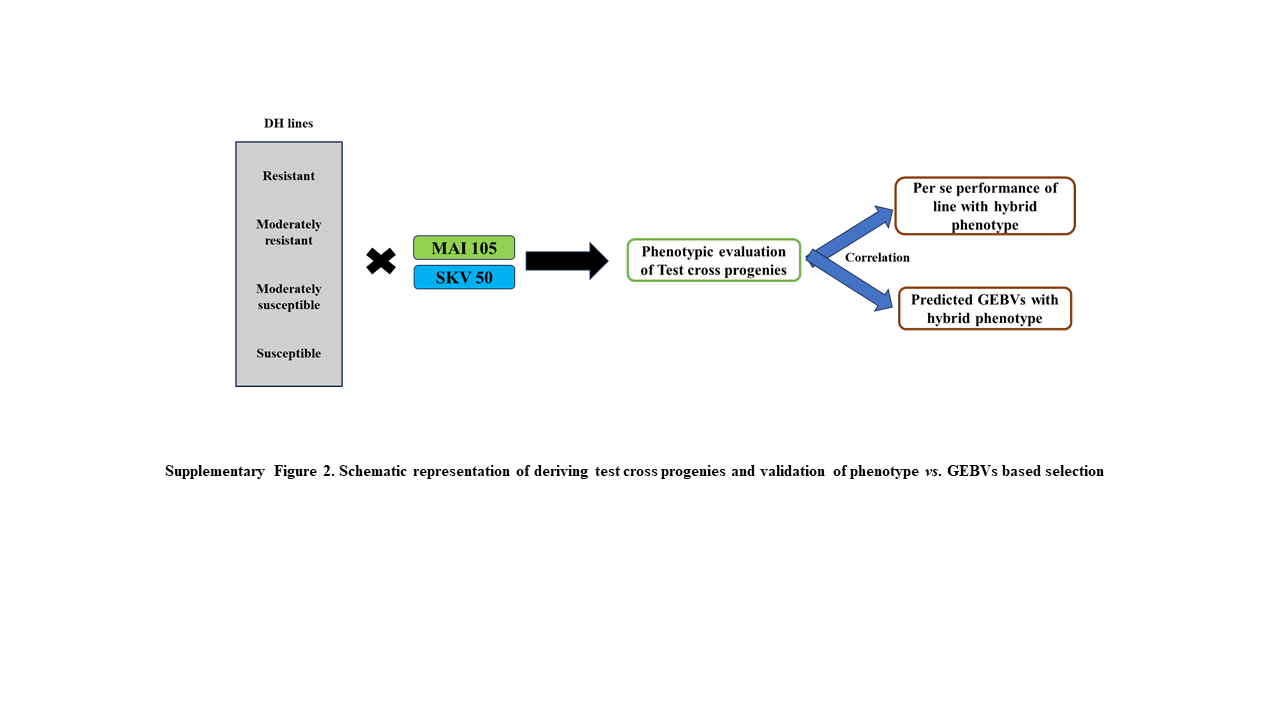

Supplement: Supplementary file 5 [file Image2.tif]

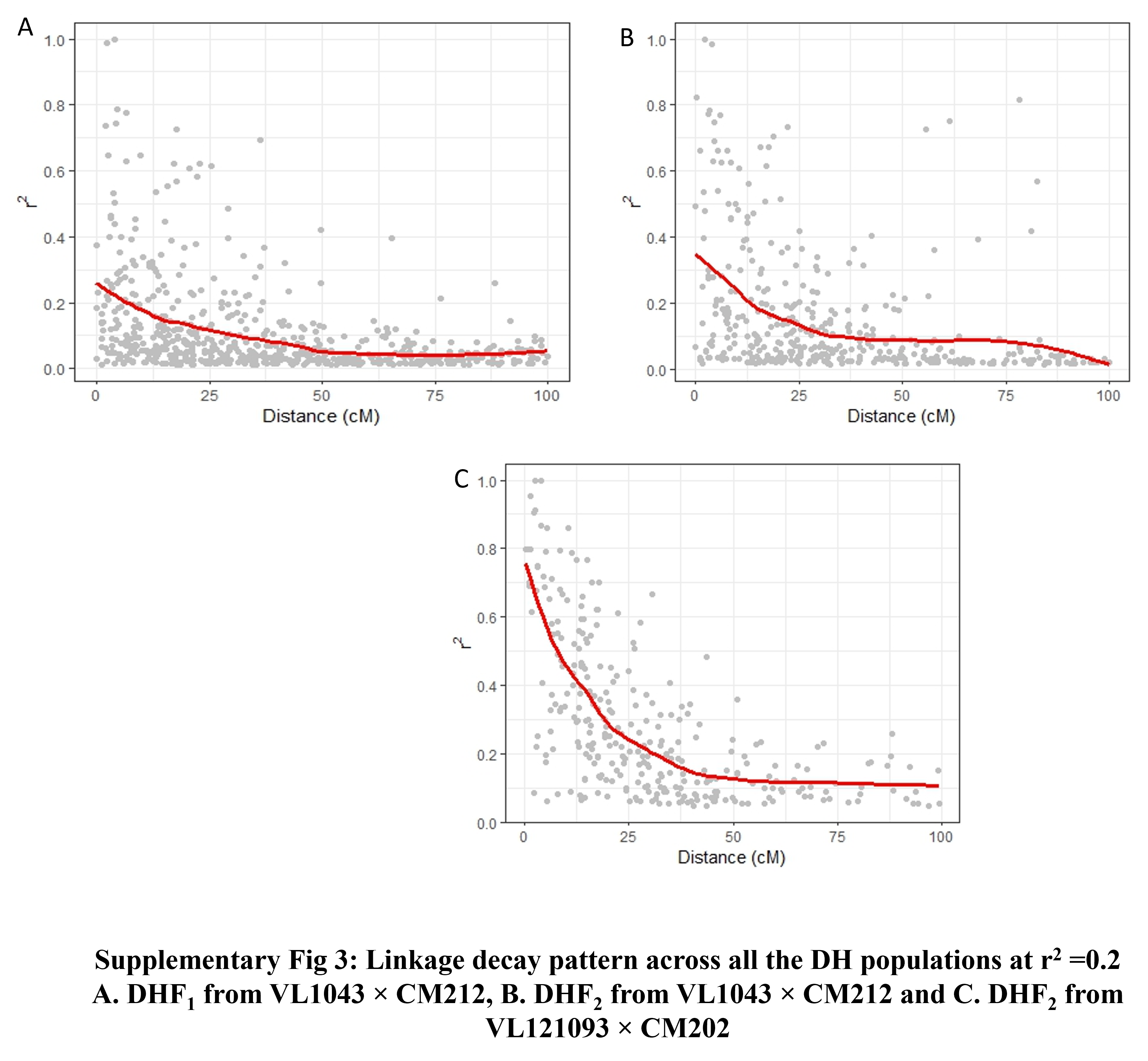

Supplement: Supplementary file 6 [file Image3.tif]

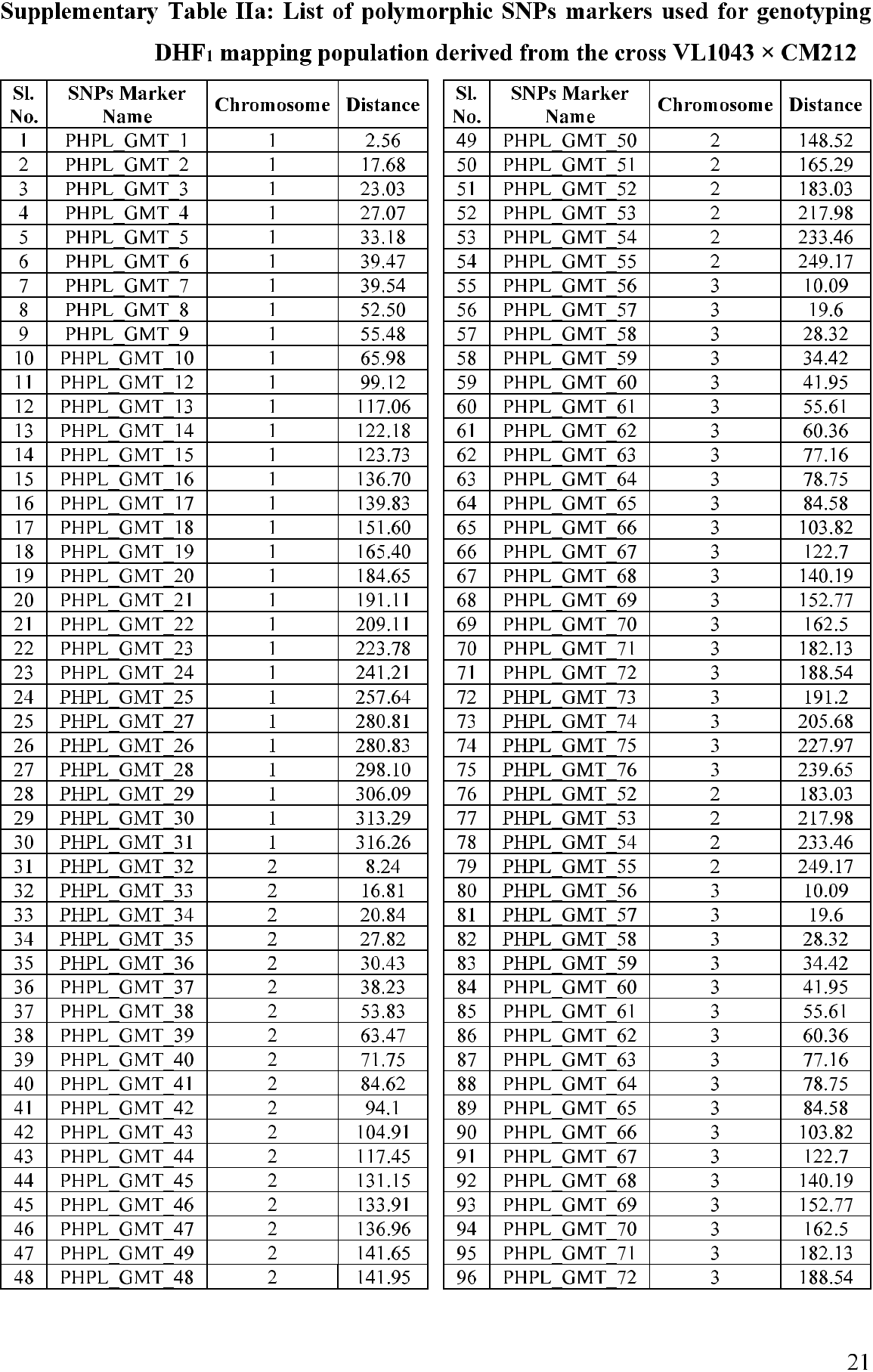


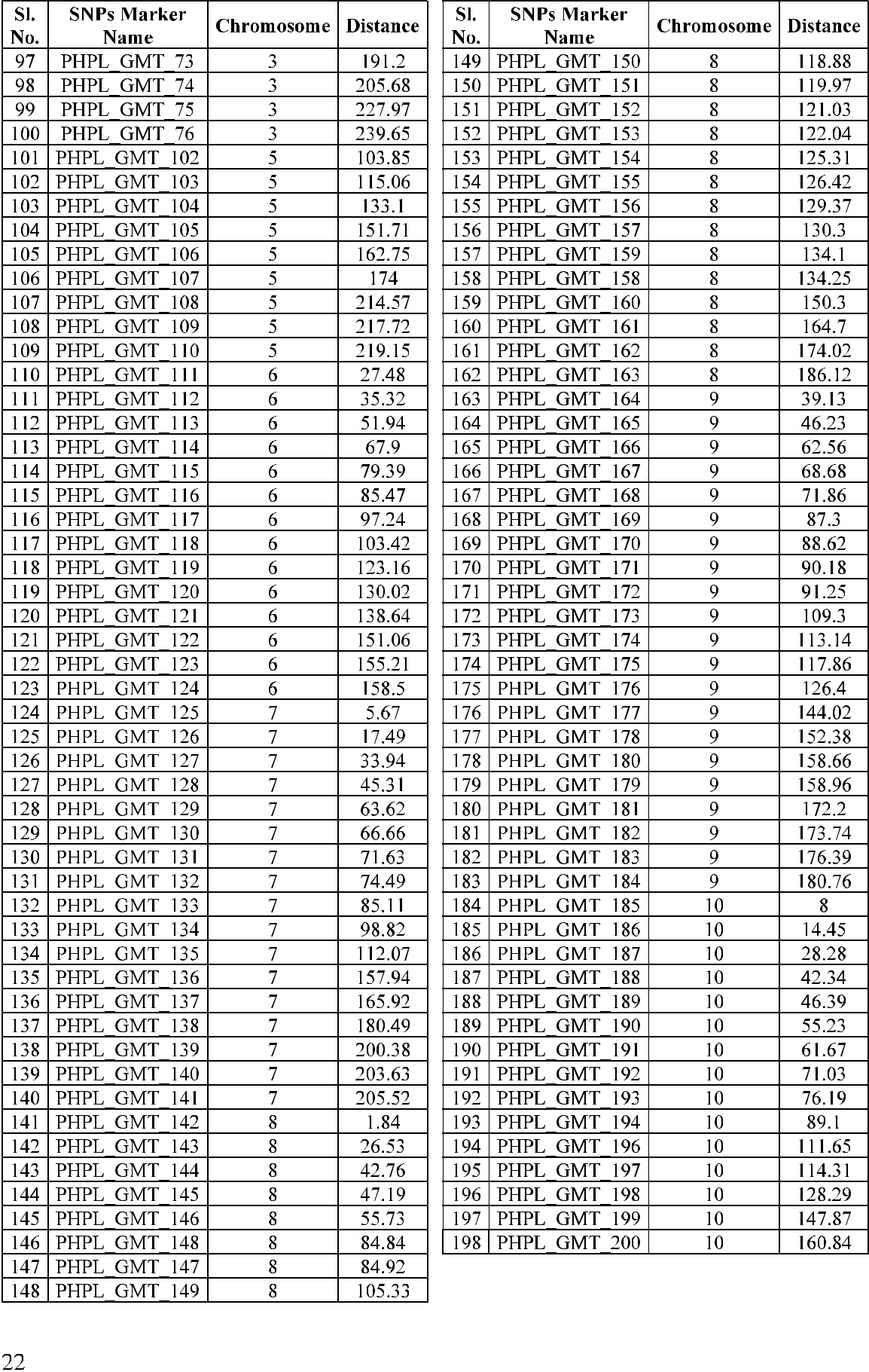


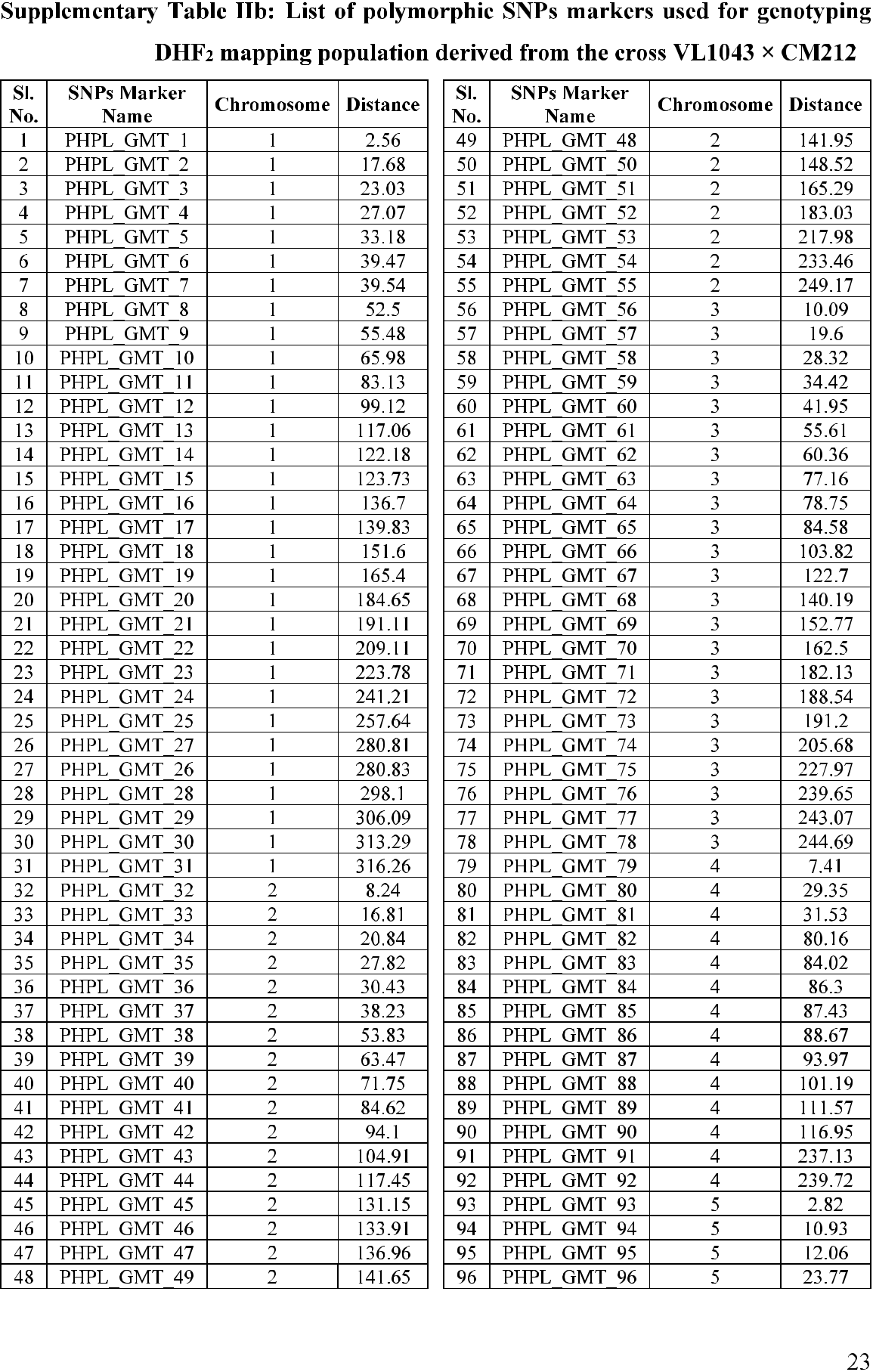


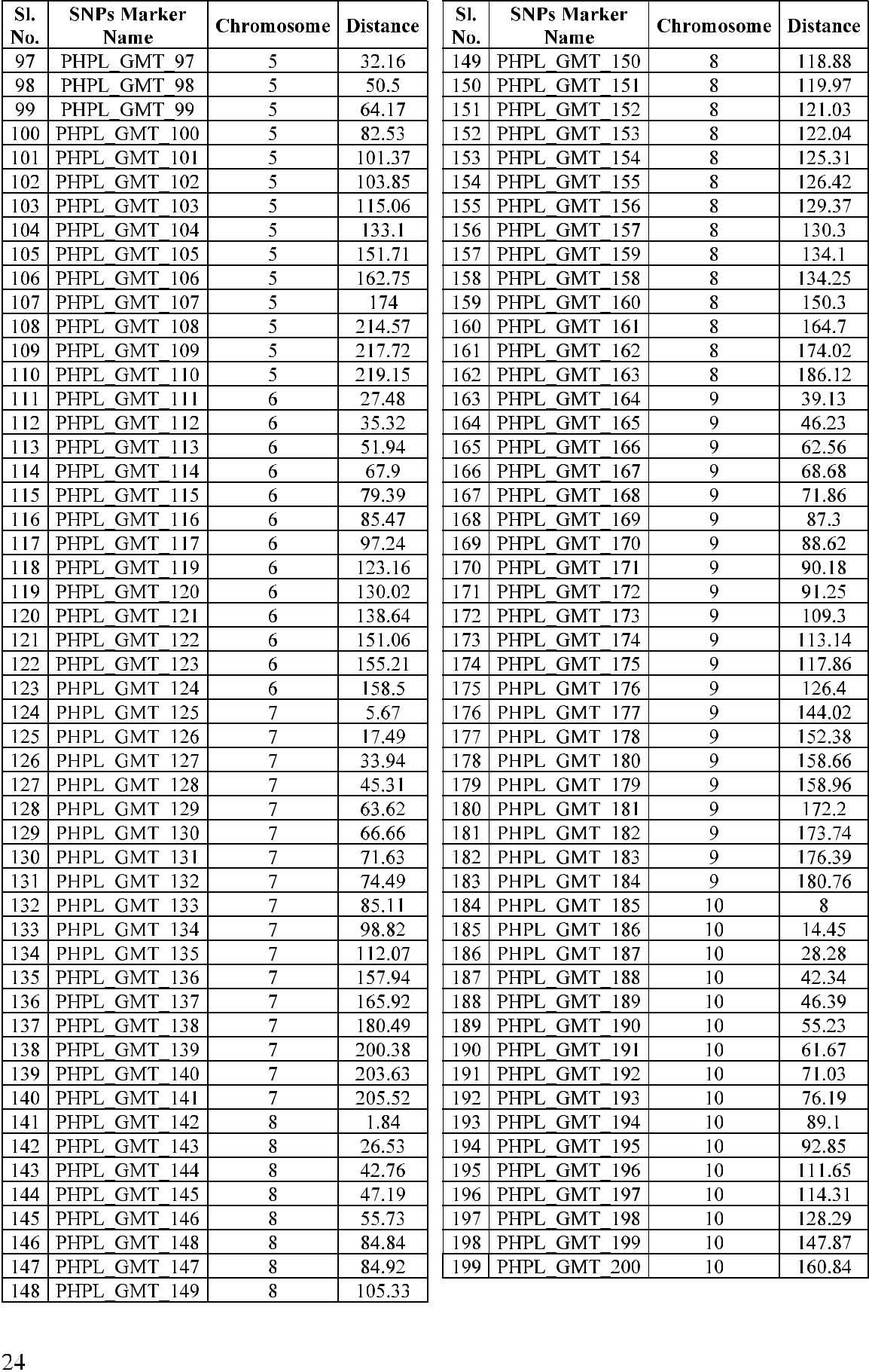


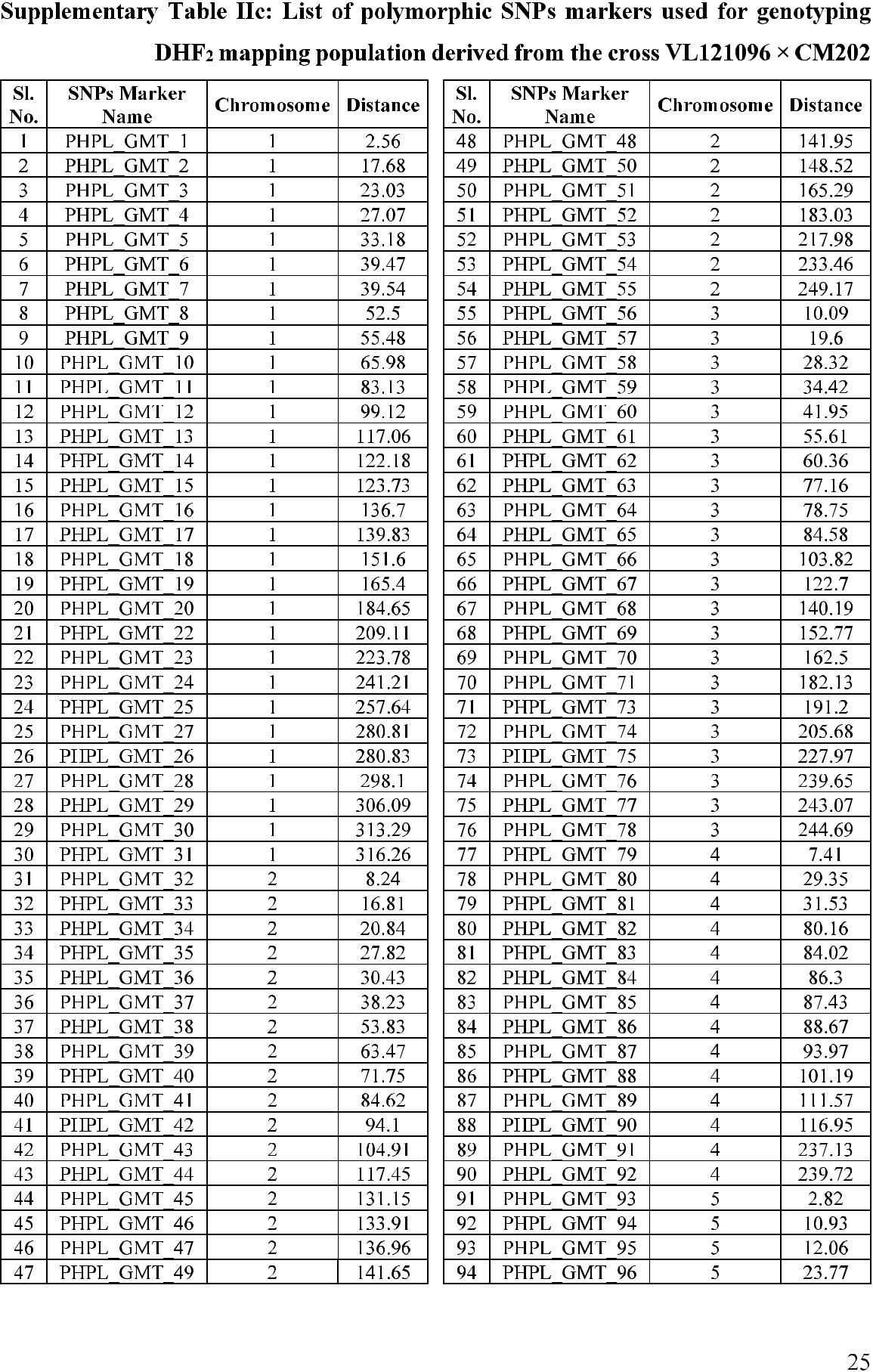

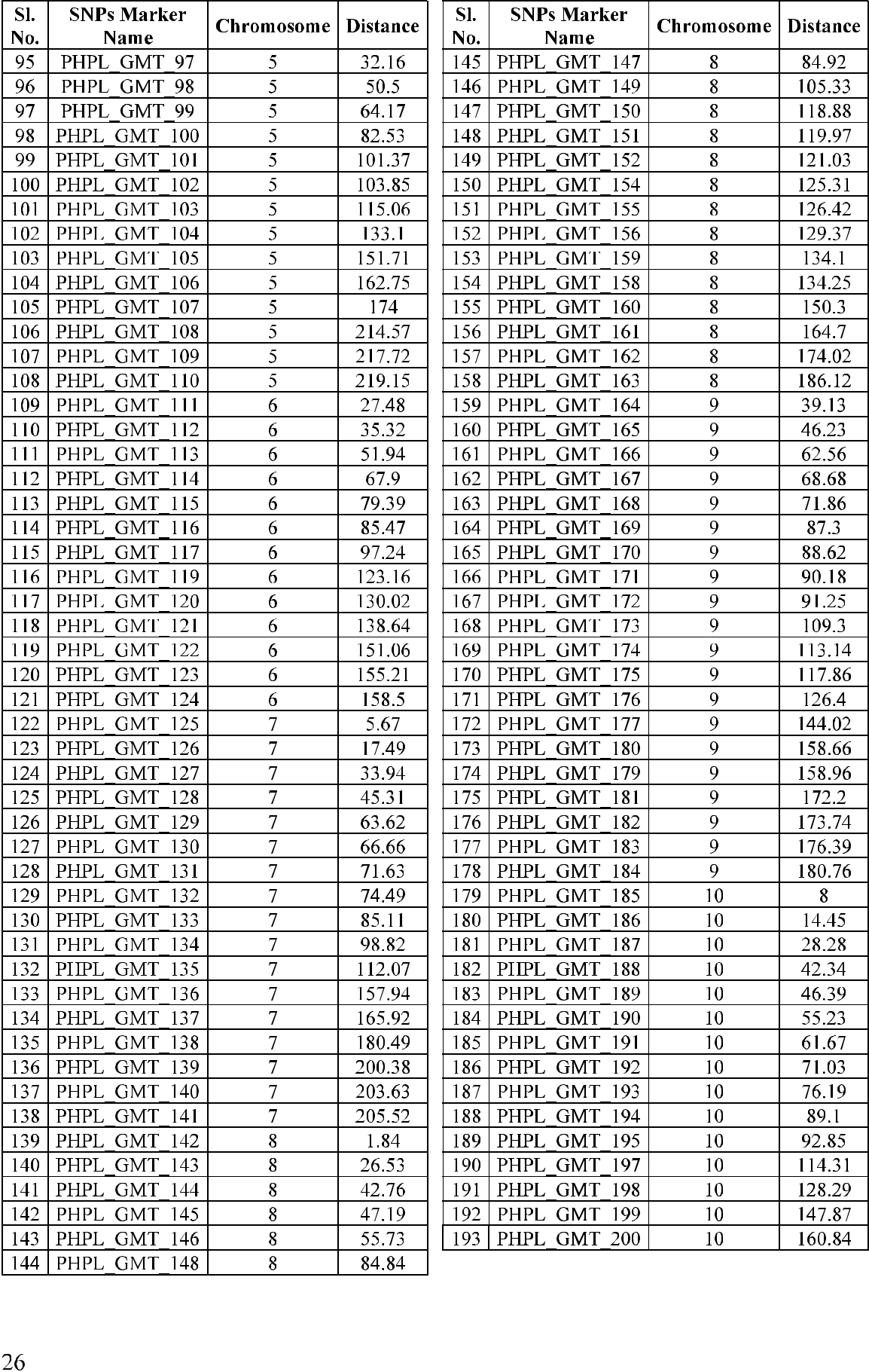

Supplement: Supplementary file 9 [file Table3.doc]
