# Supplementary material for: Assessment of the potential for genomic selection to improve resistance to fusarium stalk rot in maize
Source: Front Plant Sci. 2025 Sep 23;16:1631408. doi: 10.3389/fpls.2025.1631408 (PMC12500717; doi:10.3389/fpls.2025.1631408)
Supplement: Supplementary file 8 [file Table2.docx]

| **DH progenies**  Supplementary Table 1a: Mean disease scores for FSR across three seasons for DH population derived from F1 of VL1043 × CM212 | **WS_2018-19** | **RS_2019** | **WS_2019-20** | **Mean disease score** |
| --- | --- | --- | --- | --- |
| 1 | 4.17 | 5.63 | 4.01 | 4.60 |
| 2 | 4.37 | 3.75 | 5.31 | 4.48 |
| 3 | 4.99 | 3.39 | 4.04 | 4.14 |
| 4 | 4.58 | 4.79 | 3.57 | 4.31 |
| 5 | 4.66 | 4.46 | 4.04 | 4.39 |
| 6 | 5.66 | 3.51 | 4.35 | 4.51 |
| 7 | 3.34 | 4.12 | 7.94 | 5.13 |
| 8 | 3.84 | 4.17 | 4.04 | 4.02 |
| 9 | 4.99 | 7.85 | 4.04 | 5.63 |
| 10 | 4.58 | 4.3 | 4.24 | 4.37 |
| 11 | 7.47 | 4.15 | 3.54 | 5.05 |
| 12 | 5.82 | 4.05 | 3.92 | 4.60 |
| 13 | 5.82 | 4.3 | 4.04 | 4.72 |
| 14 | 4.5 | 4.05 | 4.37 | 4.31 |
| 15 | 4.17 | 4.1 | 4.04 | 4.10 |
| 16 | 4.17 | 4.55 | 5.4 | 4.71 |
| 17 | 4.58 | 4.05 | 4.72 | 4.45 |
| 18 | 4 | 4.09 | 3.85 | 3.98 |
| 19 | 4.58 | 4.15 | 4.98 | 4.57 |
| 20 | 6.37 | 3.84 | 3.54 | 4.58 |
| 21 | 5.2 | 4.13 | 4.09 | 4.47 |
| 22 | 4.17 | 4.05 | 4.23 | 4.15 |
| 23 | 5.66 | 4.15 | 5.95 | 5.25 |
| 24 | 3.34 | 4.23 | 5.85 | 4.47 |
| 25 | 4.17 | 4.46 | 4.06 | 4.23 |
| 26 | 4.33 | 4.57 | 5.89 | 4.93 |
| 27 | 6.98 | 3.92 | 6.13 | 5.68 |
| 28 | 6.65 | 3.98 | 4.20 | 4.94 |
| 29 | 5.55 | 4.13 | 4.16 | 4.61 |
| 30 | 7.20 | 4.11 | 4.06 | 5.12 |
| 31 | 7.47 | 2.98 | 5.16 | 5.20 |
| 32 | 4.17 | 4.73 | 3.94 | 4.28 |
| 33 | 4.17 | 4.48 | 4.06 | 4.24 |
| 34 | 4.17 | 4.33 | 6.81 | 5.10 |
| 35 | 4.58 | 3.98 | 4.21 | 4.26 |
| 36 | 7.47 | 5.82 | 4.14 | 5.81 |
| 37 | 6.92 | 4.28 | 4.94 | 5.38 |
| 38 | 7.20 | 4.15 | 4.39 | 5.25 |
| 39 | 4.17 | 4.73 | 4.06 | 4.32 |
| 40 | 4.17 | 4.23 | 4.06 | 4.15 |
| 41 | 4.99 | 4.07 | 4.17 | 4.41 |
| 42 | 4.50 | 3.98 | 4.07 | 4.18 |
| 43 | 3.96 | 4.19 | 3.80 | 3.98 |
| 44 | 5.27 | 4.20 | 4.06 | 4.51 |
| 45 | 4.58 | 3.86 | 4.32 | 4.25 |
| **DH progenies** | **WS_2018-19** | **RS_2019** | **WS_2019-20** | **Mean disease score** |
| 46 | 4.00 | 3.98 | 4.39 | 4.12 |
| 47 | 3.84 | 4.29 | 4.66 | 4.26 |
| 48 | 5.41 | 3.73 | 4.15 | 4.43 |
| 49 | 4.33 | 4.18 | 4.06 | 4.19 |
| 50 | 4.99 | 4.42 | 4.10 | 4.50 |
| 51 | 4.99 | 4.04 | 4.51 | 4.51 |
| 52 | 3.96 | 4.39 | 4.31 | 4.22 |
| 53 | 4.17 | 4.48 | 4.45 | 4.37 |
| 54 | 5.82 | 3.98 | 3.89 | 4.56 |
| 55 | 4.50 | 4.24 | 4.89 | 4.54 |
| 56 | 4.58 | 4.42 | 3.93 | 4.31 |
| 57 | 4.83 | 4.03 | 4.41 | 4.42 |
| 58 | 4.50 | 4.32 | 5.69 | 4.84 |
| 59 | 4.58 | 3.96 | 5.15 | 4.56 |
| 60 | 3.89 | 5.43 | 4.84 | 4.72 |
| 61 | 4.99 | 5.44 | 4.18 | 4.87 |
| 62 | 4.17 | 4.32 | 5.69 | 4.73 |
| 63 | 4.99 | 4.16 | 5.09 | 4.75 |
| 64 | 5.41 | 5.01 | 4.64 | 5.02 |
| 65 | 4.44 | 3.91 | 4.19 | 4.18 |
| 66 | 6.23 | 3.91 | 3.89 | 4.68 |
| 67 | 6.15 | 3.76 | 4.04 | 4.65 |
| 68 | 5.41 | 4.02 | 4.72 | 4.72 |
| 69 | 4.79 | 4.22 | 3.99 | 4.33 |
| 70 | 6.81 | 4.24 | 4.38 | 5.14 |
| 71 | 4.17 | 3.91 | 5.59 | 4.56 |
| 72 | 5.27 | 4.1 | 4.64 | 4.67 |
| 73 | 4.33 | 4.12 | 4.74 | 4.40 |
| 74 | 4.83 | 4.08 | 3.82 | 4.24 |
| 75 | 6.81 | 4.11 | 3.89 | 4.94 |
| 76 | 5.27 | 3.78 | 4.66 | 4.57 |
| 77 | 5.41 | 4.41 | 4.24 | 4.69 |
| 78 | 4.17 | 4.66 | 5.61 | 4.81 |
| 79 | 4.17 | 4.01 | 4.14 | 4.11 |
| 80 | 4.83 | 4.16 | 3.80 | 4.26 |
| 81 | 4.66 | 4.52 | 5.08 | 4.75 |
| 82 | 6.81 | 5.11 | 4.50 | 5.47 |
| 83 | 4.44 | 5.21 | 4.16 | 4.60 |
| 84 | 4.17 | 4.05 | 3.20 | 3.81 |
| 85 | 4.99 | 4.05 | 5.76 | 4.93 |
| 86 | 4.99 | 4.45 | 5.1 | 4.85 |
| 87 | 5.82 | 6.25 | 4.00 | 5.36 |
| 88 | 4.33 | 4.85 | 4.25 | 4.48 |
| 89 | 5.27 | 4.51 | 4.88 | 4.89 |
| 90 | 5.32 | 5.33 | 4.37 | 5.01 |
| **DH progenies** | **WS_2018-19** | **RS_2019** | **WS_2019-20** | **Mean disease score** |
| 91 | 5.27 | 4.62 | 4.67 | 4.85 |
| 92 | 4.58 | 4.3 | 4.00 | 4.29 |
| 93 | 5.16 | 4.79 | 4.94 | 4.96 |
| 94 | 4.58 | 4.33 | 5.03 | 4.65 |
| 95 | 4.66 | 4.44 | 5.10 | 4.73 |
| 96 | 4.17 | 4.32 | 4.13 | 4.21 |
| 97 | 5.41 | 4.26 | 3.71 | 4.46 |
| 98 | 5.66 | 4.32 | 5.44 | 5.14 |
| 99 | 5.82 | 4.05 | 4.40 | 4.76 |
| 100 | 4.17 | 4.61 | 4.00 | 4.26 |
| 101 | 6.37 | 4.19 | 4.57 | 5.04 |
| 102 | 6.09 | 4.49 | 5.55 | 5.38 |
| 103 | 4.72 | 5.19 | 5.25 | 5.05 |
| 104 | 3.61 | 4.65 | 4.85 | 4.37 |
| 105 | 4.17 | 4.30 | 5.86 | 4.78 |
| 106 | 6.23 | 4.96 | 4.15 | 5.11 |
| 107 | 6.03 | 4.97 | 3.53 | 4.84 |
| 108 | 4.83 | 4.61 | 3.79 | 4.41 |
| 109 | 6.48 | 4.03 | 4.29 | 4.93 |
| 110 | 3.96 | 3.47 | 5.22 | 4.22 |
| 111 | 6.03 | 3.97 | 3.96 | 4.65 |
| 112 | 4.17 | 5.17 | 4.46 | 4.60 |
| 113 | 7.14 | 4.37 | 3.96 | 5.16 |
| 114 | 5.27 | 4.4 | 6.06 | 5.24 |
| 115 | 4.58 | 4.34 | 4.51 | 4.48 |
| 116 | 4.99 | 4.18 | 3.47 | 4.21 |
| 117 | 4.83 | 4.07 | 4.26 | 4.39 |
| 118 | 7.47 | 3.99 | 3.96 | 5.14 |
| 119 | 4.99 | 4.07 | 4.46 | 4.51 |
| 120 | 3.34 | 4.05 | 5.29 | 4.23 |
| 121 | 4.58 | 5.38 | 4.29 | 4.75 |
| 122 | 3.89 | 3.97 | 3.96 | 3.94 |
| 123 | 3.84 | 4.9 | 3.96 | 4.23 |
| 124 | 4.79 | 4.77 | 4.34 | 4.63 |
| 125 | 3.75 | 5.10 | 3.96 | 4.27 |
| 126 | 7.14 | 4.73 | 4.62 | 5.50 |
| 127 | 4.99 | 3.97 | 4.98 | 4.65 |
| 128 | 4.17 | 3.97 | 3.75 | 3.96 |
| 129 | 4.17 | 4.22 | 3.96 | 4.12 |
| 130 | 5.27 | 4.05 | 3.83 | 4.38 |
| 131 | 5.66 | 4.13 | 4.53 | 4.77 |
| 132 | 3.75 | 3.92 | 3.96 | 3.88 |
| 133 | 4.17 | 4.31 | 3.96 | 4.15 |
| 134 | 4.00 | 4.25 | 3.84 | 4.03 |
| 135 | 4.83 | 3.48 | 3.96 | 4.09 |
| **DH progenies** | **WS_2018-19** | **RS_2019** | **WS_2019-20** | **Mean disease score** |
| 136 | 5.82 | 4.47 | 4.03 | 4.77 |
| 137 | 4.58 | 3.98 | 3.96 | 4.17 |
| 138 | 4.99 | 4.12 | 4.49 | 4.53 |
| 139 | 4.44 | 3.69 | 2.84 | 3.66 |
| 140 | 4.17 | 4.79 | 4.13 | 4.36 |
| 141 | 4.37 | 4.60 | 3.96 | 4.31 |
| 142 | 4.37 | 4.31 | 6.63 | 5.10 |
| 143 | 4.17 | 3.98 | 5.04 | 4.40 |
| 144 | 4.17 | 6.48 | 4.26 | 4.97 |
| 145 | 4.79 | 5.64 | 4.16 | 4.86 |
| 146 | 5.82 | 3.98 | 3.96 | 4.59 |
| 147 | 4.58 | 4.36 | 3.84 | 4.26 |
| 148 | 4.66 | 4.73 | 4.71 | 4.70 |
| 149 | 3.55 | 4.49 | 3.96 | 4.00 |
| 150 | 4.17 | 3.98 | 4.96 | 4.37 |
| 151 | 5.27 | 4.10 | 4.13 | 4.50 |
| 152 | 4.99 | 4.98 | 4.61 | 4.86 |
| 153 | 4.37 | 4.31 | 4.98 | 4.55 |
| 154 | 4.99 | 3.95 | 3.79 | 4.24 |
| 155 | 4.17 | 5.38 | 5.71 | 5.09 |
| 156 | 4.17 | 4.08 | 4.16 | 4.14 |
| 157 | 5.13 | 4.48 | 3.96 | 4.52 |
| 158 | 4.72 | 4.6 | 4.76 | 4.69 |
| 159 | 6.65 | 5.98 | 3.57 | 5.40 |
| 160 | 7.27 | 5.37 | 3.40 | 5.35 |
| 161 | 4.17 | 3.86 | 3.90 | 3.98 |
| 162 | 4.33 | 3.86 | 4.40 | 4.20 |
| 163 | 3.61 | 3.99 | 5.12 | 4.24 |
| 164 | 3.34 | 4.43 | 5.15 | 4.31 |
| 165 | 5.49 | 5.13 | 4.07 | 4.90 |
| 166 | 4.44 | 5.36 | 4.60 | 4.80 |
| 167 | 5.13 | 4.20 | 4.03 | 4.45 |
| 168 | 4.99 | 4.26 | 3.75 | 4.33 |
| 169 | 4.17 | 3.95 | 3.9 | 4.01 |
| 170 | 4.99 | 4.06 | 4.19 | 4.41 |
| 171 | 4.58 | 3.86 | 4.06 | 4.17 |
| 172 | 3.75 | 4.16 | 3.76 | 3.89 |
| 173 | 4.17 | 3.93 | 3.90 | 4.00 |
| 174 | 5.82 | 3.72 | 6.12 | 5.22 |
| 175 | 4.37 | 4.54 | 3.90 | 4.27 |
| 176 | 4.44 | 5.79 | 3.90 | 4.71 |
| 177 | 4.58 | 5.36 | 3.90 | 4.61 |
| 178 | 6.23 | 3.75 | 4.90 | 4.96 |
| 179 | 5.61 | 3.86 | 4.10 | 4.52 |
| 180 | 5.41 | 4.86 | 3.79 | 4.69 |
| **DH progenies** | **WS_2018-19** | **RS_2019** | **WS_2019-20** | **Mean disease score** |
| 181 | 4.17 | 4.09 | 4.07 | 4.11 |
| 182 | 4.99 | 3.84 | 4.10 | 4.31 |
| 183 | 4.17 | 4.09 | 7.11 | 5.12 |
| 184 | 3.74 | 5.20 | 3.83 | 4.26 |
| 185 | 4.33 | 7.16 | 4.35 | 5.28 |
| 186 | 4.17 | 4.11 | 5.33 | 4.54 |
| 187 | 7.07 | 4.31 | 3.90 | 5.09 |
| 188 | 3.84 | 8.21 | 3.84 | 5.30 |
| 189 | 4.38 | 4.86 | 5.58 | 4.94 |
| 190 | 8.03 | 4.07 | 3.84 | 5.31 |
| 191 | 4.17 | 4.42 | 4.09 | 4.23 |
| 192 | 4.17 | 4.17 | 5.45 | 4.60 |
| 193 | 4.44 | 3.74 | 4.69 | 4.29 |
| 194 | 4.44 | 4.30 | 3.84 | 4.19 |
| 195 | 4.58 | 4.80 | 3.84 | 4.41 |
| 196 | 6.37 | 4.13 | 4.96 | 5.15 |
| 197 | 4.99 | 4.20 | 4.19 | 4.46 |
| 198 | 6.09 | 4.99 | 4.53 | 5.20 |
| 199 | 4.79 | 4.45 | 4.14 | 4.46 |
| 200 | 4.99 | 4.79 | 4.24 | 4.67 |
| 201 | 4.17 | 4.14 | 3.84 | 4.05 |
| 202 | 4.44 | 4.74 | 4.12 | 4.43 |
| 203 | 4.33 | 3.74 | 3.84 | 3.97 |
| 204 | 4.17 | 4.01 | 4.09 | 4.09 |
| 205 | 4.17 | 4.57 | 3.64 | 4.13 |
| 206 | 4.50 | 4.34 | 3.17 | 4.00 |
| 207 | 4.44 | 3.86 | 3.84 | 4.05 |
| 208 | 3.61 | 3.85 | 3.87 | 3.78 |
| 209 | 4.17 | 3.78 | 5.52 | 4.49 |
| 210 | 6.23 | 3.80 | 4.54 | 4.86 |
| 211 | 5.96 | 4.11 | 4.85 | 4.97 |
| 212 | 4.17 | 4.34 | 4.19 | 4.23 |
| 213 | 6.65 | 3.97 | 4.39 | 5.00 |
| 214 | 4.58 | 4.13 | 4.19 | 4.30 |
| 215 | 5.41 | 4.25 | 4.34 | 4.67 |
| 216 | 4.58 | 4.63 | 4.19 | 4.47 |
| 217 | 4.44 | 3.63 | 4.40 | 4.16 |
| 218 | 6.37 | 4.44 | 4.19 | 5.00 |
| 219 | 4.83 | 4.07 | 4.85 | 4.58 |
| 220 | 3.89 | 4.23 | 5.81 | 4.64 |
| 221 | 4.58 | 3.13 | 4.24 | 3.98 |
| 222 | 4.17 | 4.22 | 4.94 | 4.44 |
| 223 | 6.09 | 4.23 | 4.28 | 4.87 |
| 224 | 6.79 | 4.44 | 4.01 | 5.08 |
| 225 | 4.17 | 4.32 | 4.19 | 4.23 |
| **DH progenies** | **WS_2018-19** | **RS_2019** | **WS_2019-20** | **Mean disease score** |
| 226 | 5.16 | 4.45 | 4.85 | 4.82 |
| 227 | 4.17 | 4.22 | 4.19 | 4.19 |
| 228 | 3.61 | 4.13 | 4.06 | 3.93 |
| 229 | 3.75 | 4.17 | 4.33 | 4.08 |
| 230 | 5.27 | 4.13 | 4.19 | 4.53 |
| 231 | 2.51 | 4.72 | 4.19 | 3.81 |
| 232 | 4.79 | 4.29 | 5.51 | 4.86 |
| 233 | 4.44 | 4.13 | 3.62 | 4.06 |
| 234 | 4.99 | 4.80 | 5.46 | 5.08 |
| 235 | 4.17 | 4.17 | 5.11 | 4.48 |
| 236 | 5.96 | 3.87 | 4.96 | 4.93 |
| 237 | 3.34 | 4.16 | 5.76 | 4.42 |
| 238 | 6.65 | 4.46 | 6.67 | 5.93 |
| 239 | 5.82 | 4.65 | 5.96 | 5.48 |
| 240 | 5.32 | 4.69 | 3.76 | 4.59 |
| 241 | 6.48 | 4.25 | 4.34 | 5.02 |
| 242 | 7.97 | 3.96 | 4.21 | 5.38 |
| 243 | 7.14 | 4.10 | 5.66 | 5.63 |
| 244 | 5.66 | 4.11 | 5.51 | 5.09 |
| 245 | 7.06 | 5.09 | 4.75 | 5.63 |
| 246 | 4.37 | 3.96 | 4.67 | 4.33 |
| 247 | 5.96 | 4.85 | 6.03 | 5.61 |
| 248 | 6.65 | 4.07 | 5.21 | 5.31 |
| 249 | 4.65 | 4.68 | 4.26 | 4.53 |
| 250 | 5.16 | 4.36 | 4.38 | 4.63 |
| 251 | 6.92 | 4.02 | 4.09 | 5.01 |
| 252 | 5.82 | 4.31 | 4.62 | 4.92 |
| 253 | 5.66 | 4.13 | 4.23 | 4.67 |
| 254 | 4.50 | 4.19 | 4.26 | 4.32 |
| 255 | 4.17 | 4.07 | 4.73 | 4.32 |
| 256 | 4.99 | 3.93 | 4.53 | 4.48 |
| 257 | 4.44 | 4.36 | 4.01 | 4.27 |
| 258 | 5.41 | 3.80 | 5.61 | 4.94 |
| 259 | 5.20 | 4.51 | 4.44 | 4.72 |
| 260 | 5.16 | 4.16 | 4.17 | 4.50 |
| 261 | 6.09 | 4.26 | 4.37 | 4.91 |
| 262 | 6.65 | 4.12 | 4.17 | 4.98 |
| 263 | 4.66 | 4.71 | 4.30 | 4.56 |
| 264 | 4.99 | 6.17 | 4.67 | 5.28 |
| 265 | 3.34 | 5.63 | 5.13 | 4.70 |
| 266 | 5.68 | 4.84 | 4.17 | 4.90 |
| 267 | 5.49 | 5.03 | 4.17 | 4.90 |
| 268 | 4.44 | 4.52 | 4.87 | 4.61 |
| 269 | 5.16 | 4.32 | 4.17 | 4.55 |
| 270 | 4.17 | 4.32 | 4.17 | 4.22 |
| **DH progenies** | **WS_2018-19** | **RS_2019** | **WS_2019-20** | **Mean disease score** |
| 271 | 4.17 | 4.11 | 4.00 | 4.09 |
| 272 | 5.32 | 4.32 | 3.17 | 4.27 |
| 273 | 5.55 | 4.32 | 5.89 | 5.25 |
| 274 | 3.89 | 4.48 | 4.43 | 4.27 |
| 275 | 4.44 | 4.75 | 4.17 | 4.45 |
| 276 | 4.79 | 4.35 | 4.17 | 4.44 |
| 277 | 6.65 | 4.82 | 4.17 | 5.21 |
| 278 | 4.58 | 4.82 | 4.22 | 4.54 |
| 279 | 4.17 | 4.63 | 4.60 | 4.47 |
| 280 | 4.66 | 4.42 | 4.00 | 4.36 |
| 281 | 5.82 | 4.32 | 4.17 | 4.77 |
| 282 | 4.17 | 4.25 | 4.95 | 4.46 |
| 283 | 4.99 | 4.52 | 4.17 | 4.56 |
| 284 | 4.17 | 4.13 | 4.50 | 4.27 |
| 285 | 4.37 | 5.16 | 5.59 | 5.04 |
| 286 | 4.54 | 4.86 | 3.59 | 4.33 |
| 287 | 4.17 | 6.34 | 5.71 | 5.41 |
| 288 | 4.99 | 4.55 | 7.54 | 5.69 |
| 289 | 6.65 | 4.08 | 4.43 | 5.05 |
| 290 | 4.17 | 7.25 | 4.93 | 5.45 |
| 291 | 7.06 | 5.01 | 4.18 | 5.42 |
| 292 | 7.47 | 4.01 | 3.93 | 5.14 |
| 293 | 4.17 | 4.51 | 3.93 | 4.20 |
| 294 | 4.44 | 4.39 | 4.20 | 4.34 |
| 295 | 4.17 | 4.01 | 3.59 | 3.92 |
| 296 | 4.99 | 4.16 | 3.93 | 4.36 |
| 297 | 6.37 | 4.06 | 3.93 | 4.79 |
| 298 | 4.50 | 4.52 | 3.93 | 4.32 |
| 299 | 3.75 | 4.22 | 4.26 | 4.08 |
| 300 | 5.49 | 3.94 | 3.93 | 4.45 |
| 301 | 4.99 | 4.55 | 4.81 | 4.78 |
| 302 | 4.37 | 4.32 | 3.93 | 4.21 |
| 303 | 6.92 | 4.44 | 6.08 | 5.81 |
| 304 | 7.06 | 4.81 | 5.87 | 5.91 |
| 305 | 5.27 | 7.81 | 5.22 | 6.10 |
| 306 | 4.58 | 6.23 | 3.78 | 4.86 |
| 307 | 5.55 | 4.60 | 4.12 | 4.76 |
| 308 | 5.82 | 4.26 | 3.93 | 4.67 |
| 309 | 7.47 | 4.16 | 3.93 | 5.19 |
| 310 | 4.37 | 4.01 | 4.48 | 4.29 |
| 311 | 4.17 | 4.01 | 4.54 | 4.24 |
| 312 | 4.22 | 3.88 | 5.39 | 4.50 |
| 313 | 4.17 | 4.99 | 4.17 | 4.44 |
| 314 | 4.99 | 5.68 | 4.15 | 4.94 |
| 315 | 4.17 | 4.25 | 4.12 | 4.18 |
| **DH progenies** | **WS_2018-19** | **RS_2019** | **WS_2019-20** | **Mean disease score** |
| 316 | 4.99 | 4.21 | 5.09 | 4.76 |
| 317 | 4.17 | 4.14 | 4.07 | 4.13 |
| 318 | 4.17 | 4.42 | 5.07 | 4.55 |
| 319 | 4.17 | 4.19 | 4.06 | 4.14 |
| 320 | 5.55 | 4.04 | 5.10 | 4.90 |
| 321 | 4.58 | 4.35 | 4.23 | 4.39 |
| 322 | 5.82 | 4.10 | 4.23 | 4.72 |
| 323 | 3.89 | 4.13 | 4.07 | 4.03 |
| 324 | 5.82 | 4.21 | 4.07 | 4.70 |
| 325 | 4.37 | 4.10 | 4.02 | 4.16 |
| 326 | 6.15 | 3.99 | 4.07 | 4.74 |
| 327 | 4.83 | 4.29 | 4.07 | 4.40 |
| 328 | 5.20 | 4.04 | 4.52 | 4.59 |
| 329 | 3.89 | 4.69 | 4.07 | 4.22 |
| 330 | 3.34 | 4.95 | 5.48 | 4.59 |
| 331 | 4.17 | 3.88 | 3.92 | 3.99 |
| 332 | 4.17 | 4.66 | 4.07 | 4.30 |
| 333 | 4.99 | 4.14 | 4.15 | 4.43 |
| 334 | 5.99 | 4.34 | 5.85 | 5.39 |
| 335 | 3.96 | 4.14 | 3.15 | 3.75 |
| 336 | 3.75 | 4.54 | 4.15 | 4.15 |

**Supplementary Table 1b: Mean disease scores for FSR across three seasons for DH population derived from F_2_ of VL1043 × CM212**

| **DH progeny No.** | **RS_2019** | **WS_2019-20** | **Mean disease score** |
| --- | --- | --- | --- |
| 1 | 5.41 | 4.26 | 4.84 |
| 2 | 5.26 | 4.19 | 4.73 |
| 3 | 4.34 | 4.23 | 4.28 |
| 4 | 4.65 | 4.06 | 4.35 |
| 5 | 4.38 | 4.06 | 4.22 |
| 6 | 4.38 | 4.06 | 4.22 |
| 7 | 3.73 | 3.90 | 3.81 |
| 8 | 3.58 | 4.06 | 3.82 |
| 9 | 2.98 | 5.44 | 4.21 |
| 10 | 3.98 | 4.78 | 4.38 |
| 11 | 4.15 | 4.29 | 4.22 |
| 12 | 2.98 | 4.46 | 3.72 |
| 13 | 3.98 | 3.56 | 3.77 |
| 14 | 4.98 | 4.81 | 4.90 |
| 15 | 3.48 | 4.23 | 3.85 |
| 16 | 4.98 | 4.40 | 4.69 |
| 17 | 4.26 | 3.06 | 3.66 |
| 18 | 4.55 | 4.31 | 4.43 |
| 19 | 4.81 | 4.06 | 4.44 |
| 20 | 4.58 | 4.06 | 4.32 |
| 21 | 4.98 | 4.90 | 4.94 |
| 22 | 3.31 | 4.06 | 3.69 |
| 23 | 3.41 | 4.52 | 3.97 |
| 24 | 4.08 | 4.30 | 4.19 |
| 25 | 4.33 | 3.85 | 4.09 |
| 26 | 6.58 | 5.45 | 6.01 |
| 27 | 5.41 | 4.16 | 4.79 |
| 28 | 4.45 | 4.45 | 4.45 |
| 29 | 5.41 | 4.52 | 4.97 |
| 30 | 5.08 | 5.39 | 5.24 |
| 31 | 5.36 | 4.59 | 4.98 |
| 32 | 4.40 | 4.02 | 4.21 |
| 33 | 4.88 | 4.35 | 4.62 |
| 34 | 4.08 | 4.22 | 4.15 |
| 35 | 4.08 | 4.77 | 4.42 |
| 36 | 3.94 | 4.73 | 4.33 |
| 37 | 5.08 | 4.19 | 4.63 |
| 38 | 4.08 | 4.32 | 4.20 |
| 39 | 3.08 | 3.82 | 3.45 |
| 40 | 3.75 | 4.35 | 4.05 |
| 41 | 4.26 | 5.25 | 4.75 |
| **DH progeny No.** | **RS_2019** | **WS_2019-20** | **Mean disease score** |
| 42 | 4.49 | 4.91 | 4.70 |
| 43 | 3.76 | 4.41 | 4.09 |
| 44 | 3.84 | 4.16 | 4.00 |
| 45 | 4.09 | 4.06 | 4.07 |
| 46 | 3.66 | 4.36 | 4.01 |
| 47 | 4.52 | 5.66 | 5.09 |
| 48 | 4.59 | 5.51 | 5.05 |
| 49 | 3.93 | 3.91 | 3.92 |
| 50 | 4.43 | 4.16 | 4.30 |
| 51 | 4.09 | 3.91 | 4.00 |
| 52 | 4.69 | 4.41 | 4.55 |
| 53 | 5.93 | 4.91 | 5.42 |
| 54 | 4.52 | 3.91 | 4.22 |
| 55 | 4.09 | 4.25 | 4.17 |
| 56 | 4.49 | 5.41 | 4.95 |
| 57 | 4.34 | 3.41 | 3.88 |
| 58 | 3.43 | 3.91 | 3.67 |
| 59 | 4.09 | 4.27 | 4.18 |
| 60 | 4.09 | 4.25 | 4.17 |
| 61 | 5.09 | 5.66 | 5.38 |
| 62 | 4.59 | 4.27 | 4.43 |
| 63 | 4.66 | 4.20 | 4.43 |
| 64 | 3.53 | 4.47 | 4.00 |
| 65 | 4.28 | 3.76 | 4.02 |
| 66 | 4.78 | 5.13 | 4.96 |
| 67 | 4.95 | 4.63 | 4.79 |
| 68 | 4.06 | 3.93 | 4.00 |
| 69 | 3.78 | 4.30 | 4.04 |
| 70 | 4.11 | 3.80 | 3.96 |
| 71 | 4.45 | 4.30 | 4.37 |
| 72 | 3.61 | 4.38 | 4.00 |
| 73 | 3.03 | 4.13 | 3.58 |
| 74 | 3.92 | 4.13 | 4.03 |
| 75 | 4.78 | 4.33 | 4.56 |
| 76 | 3.45 | 4.28 | 3.86 |
| 77 | 4.53 | 4.13 | 4.33 |
| 78 | 5.11 | 6.47 | 5.79 |
| 79 | 3.78 | 4.63 | 4.21 |
| 80 | 5.78 | 6.73 | 6.26 |
| 81 | 4.58 | 3.88 | 4.23 |
| 82 | 3.88 | 4.38 | 4.13 |
| 83 | 3.92 | 5.56 | 4.74 |
| 84 | 4.18 | 4.13 | 4.16 |
| 85 | 3.90 | 5.53 | 4.72 |
| 86 | 3.78 | 4.13 | 3.96 |
| **DH progeny No.** | **RS_2019** | **WS_2019-20** | **Mean disease score** |
| 87 | 4.65 | 3.18 | 3.92 |
| 88 | 3.90 | 3.68 | 3.79 |
| 89 | 5.90 | 6.18 | 6.04 |
| 90 | 4.12 | 4.08 | 4.10 |
| 91 | 5.76 | 5.06 | 5.41 |
| 92 | 4.15 | 4.68 | 4.42 |
| 93 | 4.07 | 3.68 | 3.87 |
| 94 | 4.23 | 3.68 | 3.96 |
| 95 | 3.90 | 4.48 | 4.19 |
| 96 | 4.90 | 3.68 | 4.29 |
| 97 | 4.40 | 4.68 | 4.54 |
| 98 | 5.40 | 4.68 | 5.04 |
| 99 | 4.40 | 5.14 | 4.77 |
| 100 | 2.90 | 2.68 | 2.79 |
| 101 | 4.23 | 5.07 | 4.65 |
| 102 | 3.90 | 3.68 | 3.79 |
| 103 | 3.90 | 3.68 | 3.79 |
| 104 | 4.23 | 3.43 | 3.83 |
| 105 | 4.18 | 4.88 | 4.53 |
| 106 | 4.90 | 4.68 | 4.79 |
| 107 | 3.90 | 3.93 | 3.92 |
| 108 | 4.41 | 4.57 | 4.49 |
| 109 | 4.16 | 3.87 | 4.02 |
| 110 | 4.04 | 5.07 | 4.56 |
| 111 | 3.91 | 4.57 | 4.24 |
| 112 | 3.06 | 4.07 | 3.56 |
| 113 | 6.66 | 5.85 | 6.26 |
| 114 | 3.75 | 4.07 | 3.91 |
| 115 | 3.91 | 4.47 | 4.19 |
| 116 | 3.91 | 4.27 | 4.09 |
| 117 | 3.91 | 4.07 | 3.99 |
| 118 | 6.16 | 7.07 | 6.62 |
| 119 | 4.02 | 5.22 | 4.62 |
| 120 | 3.80 | 4.41 | 4.10 |
| 121 | 3.91 | 5.28 | 4.60 |
| 122 | 4.91 | 4.07 | 4.49 |
| 123 | 2.20 | 3.82 | 3.01 |
| 124 | 3.91 | 4.07 | 3.99 |
| 125 | 4.21 | 5.22 | 4.71 |
| 126 | 4.25 | 4.18 | 4.22 |
| 127 | 4.58 | 4.25 | 4.42 |
| 128 | 3.91 | 4.07 | 3.99 |
| 129 | 3.58 | 2.87 | 3.23 |
| 130 | 3.56 | 3.99 | 3.78 |
| 131 | 4.16 | 3.99 | 4.08 |
| **DH progeny No.** | **RS_2019** | **WS_2019-20** | **Mean disease score** |
| 132 | 4.61 | 5.33 | 4.97 |
| 133 | 3.91 | 2.33 | 3.12 |
| 134 | 4.16 | 3.99 | 4.08 |
| 135 | 4.16 | 4.33 | 4.25 |
| 136 | 5.27 | 4.38 | 4.83 |
| 137 | 4.66 | 3.99 | 4.33 |
| 138 | 4.36 | 4.79 | 4.58 |
| 139 | 4.00 | 4.16 | 4.08 |
| 140 | 4.31 | 3.49 | 3.90 |
| 141 | 6.02 | 5.99 | 6.01 |
| 142 | 3.83 | 3.83 | 3.83 |
| 143 | 5.16 | 5.41 | 5.29 |
| 144 | 4.16 | 4.37 | 4.27 |
| 145 | 4.16 | 4.64 | 4.40 |
| 146 | 4.66 | 4.33 | 4.50 |
| 147 | 4.54 | 4.77 | 4.66 |
| 148 | 4.29 | 3.99 | 4.14 |
| 149 | 4.16 | 4.37 | 4.27 |
| 150 | 3.66 | 3.49 | 3.58 |
| 151 | 3.50 | 3.49 | 3.50 |
| 152 | 4.96 | 5.40 | 5.18 |
| 153 | 4.16 | 3.99 | 4.08 |
| 154 | 4.82 | 5.46 | 5.14 |
| 155 | 4.02 | 4.10 | 4.06 |
| 156 | 4.02 | 4.44 | 4.23 |
| 157 | 4.52 | 5.10 | 4.81 |
| 158 | 4.42 | 5.51 | 4.97 |
| 159 | 4.02 | 4.10 | 4.06 |
| 160 | 4.02 | 4.23 | 4.13 |
| 161 | 3.88 | 4.10 | 3.99 |
| 162 | 4.02 | 6.10 | 5.06 |
| 163 | 4.25 | 4.44 | 4.34 |
| 164 | 4.36 | 4.53 | 4.44 |
| 165 | 4.52 | 4.60 | 4.56 |
| 166 | 4.02 | 4.35 | 4.19 |
| 167 | 3.36 | 4.85 | 4.11 |
| 168 | 3.69 | 4.10 | 3.90 |
| 169 | 4.19 | 4.73 | 4.46 |
| 170 | 4.52 | 4.96 | 4.74 |
| 171 | 4.02 | 4.10 | 4.06 |
| 172 | 3.99 | 4.46 | 4.23 |
| 173 | 7.24 | 5.46 | 6.35 |
| 174 | 3.79 | 4.87 | 4.33 |
| 175 | 4.39 | 4.09 | 4.24 |
| 176 | 4.10 | 4.09 | 4.10 |
| **DH progeny No.** | **RS_2019** | **WS_2019-20** | **Mean disease score** |
| 177 | 4.49 | 4.26 | 4.37 |
| 178 | 4.16 | 5.26 | 4.71 |
| 179 | 4.16 | 4.09 | 4.12 |
| 180 | 4.14 | 5.09 | 4.61 |
| 181 | 4.42 | 5.76 | 5.09 |
| 182 | 4.16 | 5.59 | 4.87 |
| 183 | 4.16 | 4.09 | 4.12 |
| 184 | 3.59 | 4.09 | 3.84 |
| 185 | 3.99 | 5.26 | 4.62 |
| 186 | 3.99 | 4.42 | 4.21 |
| 187 | 4.49 | 3.80 | 4.15 |
| 188 | 4.16 | 3.09 | 3.62 |
| 189 | 4.33 | 5.07 | 4.70 |
| 190 | 3.56 | 4.21 | 3.89 |
| 191 | 3.99 | 4.09 | 4.04 |
| 192 | 3.99 | 4.66 | 4.33 |
| 193 | 3.79 | 3.59 | 3.69 |
| 194 | 4.12 | 3.92 | 4.02 |
| 195 | 3.12 | 3.92 | 3.52 |
| 196 | 4.12 | 3.92 | 4.02 |
| 197 | 4.29 | 4.32 | 4.30 |
| 198 | 4.29 | 4.49 | 4.39 |
| 199 | 3.87 | 3.92 | 3.90 |
| 200 | 4.12 | 4.57 | 4.35 |
| 201 | 4.32 | 3.92 | 4.12 |
| 202 | 4.12 | 3.67 | 3.90 |
| 203 | 4.32 | 4.70 | 4.51 |
| 204 | 4.45 | 4.67 | 4.56 |
| 205 | 4.37 | 4.59 | 4.48 |
| 206 | 4.79 | 3.52 | 4.15 |
| 207 | 4.37 | 3.78 | 4.07 |
| 208 | 4.79 | 3.92 | 4.35 |
| 209 | 4.12 | 3.92 | 4.02 |
| 210 | 4.29 | 4.59 | 4.44 |
| 211 | 3.62 | 3.92 | 3.77 |
| 212 | 4.12 | 4.21 | 4.16 |
| 213 | 4.55 | 3.92 | 4.24 |
| 214 | 5.12 | 4.42 | 4.77 |
| 215 | 4.62 | 4.92 | 4.77 |
| 216 | 3.79 | 4.58 | 4.18 |
| 217 | 4.12 | 4.09 | 4.10 |
| 218 | 4.32 | 4.32 | 4.32 |
| 219 | 3.96 | 4.98 | 4.47 |
| 220 | 4.21 | 4.15 | 4.18 |
| 221 | 4.29 | 5.48 | 4.89 |
| **DH progeny No.** | **RS_2019** | **WS_2019-20** | **Mean disease score** |
| 222 | 3.96 | 4.55 | 4.25 |
| 223 | 4.51 | 4.15 | 4.33 |
| 224 | 3.96 | 4.15 | 4.05 |
| 225 | 4.76 | 4.81 | 4.78 |
| 226 | 3.96 | 4.98 | 4.47 |
| 227 | 4.06 | 4.29 | 4.17 |
| 228 | 5.63 | 5.56 | 5.59 |
| 229 | 3.96 | 4.81 | 4.38 |
| 230 | 4.96 | 5.56 | 5.26 |
| 231 | 4.96 | 5.15 | 5.05 |
| 232 | 6.29 | 6.15 | 6.22 |
| 233 | 4.29 | 5.03 | 4.66 |
| 234 | 4.16 | 4.72 | 4.44 |
| 235 | 3.96 | 4.15 | 4.05 |
| 236 | 5.96 | 6.15 | 6.05 |
| 237 | 3.98 | 3.98 | 3.98 |
| 238 | 3.98 | 3.98 | 3.98 |
| 239 | 4.18 | 4.38 | 4.28 |
| 240 | 4.48 | 3.98 | 4.23 |
| 241 | 4.32 | 3.98 | 4.15 |
| 242 | 3.65 | 4.41 | 4.03 |
| 243 | 4.98 | 3.98 | 4.48 |
| 244 | 4.48 | 4.98 | 4.73 |
| 245 | 3.98 | 4.64 | 4.31 |
| 246 | 5.48 | 4.98 | 5.23 |
| 247 | 4.48 | 4.38 | 4.43 |
| 248 | 5.58 | 4.58 | 5.08 |
| 249 | 3.48 | 3.98 | 3.73 |
| 250 | 4.23 | 3.98 | 4.11 |
| 251 | 6.32 | 5.98 | 6.15 |
| 252 | 8.98 | 7.98 | 8.48 |
| 253 | 3.98 | 5.48 | 4.73 |
| 254 | 5.98 | 5.98 | 5.98 |
| 255 | 3.98 | 4.73 | 4.36 |
| 256 | 3.42 | 4.02 | 3.72 |
| 257 | 4.02 | 4.28 | 4.15 |
| 258 | 6.02 | 4.88 | 5.45 |
| 259 | 7.02 | 5.88 | 6.45 |
| 260 | 5.02 | 3.88 | 4.45 |
| 261 | 5.02 | 5.54 | 5.28 |
| 262 | 5.02 | 4.54 | 4.78 |
| 263 | 8.02 | 7.04 | 7.53 |
| 264 | 4.02 | 4.74 | 4.38 |
| 265 | 4.02 | 4.54 | 4.28 |
| 266 | 4.02 | 4.04 | 4.03 |
| **DH progeny No.** | **RS_2019** | **WS_2019-20** | **Mean disease score** |
| 267 | 6.02 | 6.84 | 6.43 |
| 268 | 7.02 | 7.70 | 7.36 |
| 269 | 4.02 | 4.54 | 4.28 |
| 270 | 5.02 | 5.70 | 5.36 |
| 271 | 4.27 | 4.54 | 4.40 |
| 272 | 3.35 | 4.05 | 3.70 |
| 273 | 4.02 | 3.88 | 3.95 |
| 274 | 4.02 | 4.72 | 4.37 |
| 275 | 3.02 | 4.04 | 3.53 |
| 276 | 5.69 | 5.88 | 5.78 |
| 277 | 4.02 | 4.84 | 4.43 |
| 278 | 4.02 | 4.88 | 4.45 |
| 279 | 5.02 | 4.70 | 4.86 |
| 280 | 4.22 | 4.74 | 4.48 |

**Supplementary Table 1c: Mean disease scorefor FSR across three seasons for DH population derived from F_2_ of VL121096 × CM202**

| **DH progeny No.** | **RS_2019** | **WS_2019-20** | **Mean disease score** |
| --- | --- | --- | --- |
| 1 | 3.73 | 4.07 | 3.90 |
| 2 | 4.21 | 4.27 | 4.24 |
| 3 | 3.64 | 4.07 | 3.86 |
| 4 | 4.36 | 4.57 | 4.46 |
| 5 | 3.93 | 4.73 | 4.33 |
| 6 | 3.93 | 4.07 | 4.00 |
| 7 | 3.93 | 4.07 | 4.00 |
| 8 | 5.18 | 4.57 | 4.88 |
| 9 | 4.26 | 4.32 | 4.29 |
| 10 | 3.93 | 4.57 | 4.25 |
| 11 | 4.93 | 4.57 | 4.75 |
| 12 | 4.18 | 4.57 | 4.38 |
| 13 | 3.93 | 3.90 | 3.92 |
| 14 | 4.18 | 4.57 | 4.38 |
| 15 | 4.37 | 4.07 | 4.22 |
| 16 | 4.43 | 4.07 | 4.25 |
| 17 | 3.85 | 4.40 | 4.13 |
| 18 | 4.32 | 4.57 | 4.45 |
| 19 | 4.98 | 4.82 | 4.90 |
| 20 | 4.18 | 3.07 | 3.63 |
| 21 | 4.68 | 4.07 | 4.38 |
| 22 | 4.18 | 4.57 | 4.38 |
| 23 | 4.46 | 4.92 | 4.69 |
| 24 | 5.78 | 6.57 | 6.18 |
| 25 | 6.18 | 7.07 | 6.63 |
| 26 | 7.18 | 6.74 | 6.96 |
| 27 | 4.43 | 4.67 | 4.55 |
| 28 | 4.18 | 4.07 | 4.13 |
| 29 | 4.32 | 3.73 | 4.03 |
| 30 | 3.85 | 4.12 | 3.98 |
| 31 | 4.18 | 3.95 | 4.07 |
| 32 | 4.18 | 3.82 | 4.00 |
| 33 | 3.68 | 2.37 | 3.03 |
| 34 | 3.38 | 3.62 | 3.50 |
| 35 | 4.18 | 4.12 | 4.15 |
| 36 | 4.85 | 4.62 | 4.73 |
| 37 | 5.58 | 5.12 | 5.35 |
| 38 | 4.68 | 4.62 | 4.65 |
| 39 | 3.68 | 3.62 | 3.65 |
| 40 | 4.18 | 3.87 | 4.03 |
| 41 | 4.68 | 4.62 | 4.65 |
| 42 | 3.68 | 3.62 | 3.65 |
| **DH progeny No.** | **RS_2019** | **WS_2019-20** | **Mean disease score** |
| 43 | 5.18 | 4.22 | 4.70 |
| 44 | 4.26 | 4.45 | 4.36 |
| 45 | 4.18 | 5.62 | 4.90 |
| 46 | 3.93 | 3.72 | 3.83 |
| 47 | 4.93 | 5.45 | 5.19 |
| 48 | 4.18 | 4.79 | 4.48 |
| 49 | 3.93 | 4.12 | 4.03 |
| 50 | 4.33 | 4.12 | 4.23 |
| 51 | 3.43 | 4.12 | 3.78 |
| 52 | 4.18 | 4.12 | 4.15 |
| 53 | 6.77 | 5.55 | 6.16 |
| 54 | 4.26 | 4.12 | 4.19 |
| 55 | 4.43 | 4.12 | 4.28 |
| 56 | 5.10 | 5.30 | 5.20 |
| 57 | 6.10 | 5.55 | 5.83 |
| 58 | 4.26 | 4.62 | 4.44 |
| 59 | 4.43 | 5.12 | 4.78 |
| 60 | 5.68 | 3.95 | 4.82 |
| 61 | 4.43 | 4.12 | 4.28 |
| 62 | 5.15 | 4.87 | 5.01 |
| 63 | 4.33 | 4.12 | 4.23 |
| 64 | 3.60 | 4.12 | 3.86 |
| 65 | 4.35 | 4.57 | 4.46 |
| 66 | 4.51 | 4.21 | 4.36 |
| 67 | 3.48 | 4.57 | 4.03 |
| 68 | 6.28 | 7.40 | 6.84 |
| 69 | 3.11 | 1.74 | 2.42 |
| 70 | 3.68 | 4.24 | 3.96 |
| 71 | 3.93 | 4.40 | 4.17 |
| 72 | 4.18 | 3.57 | 3.88 |
| 73 | 3.68 | 4.07 | 3.88 |
| 74 | 3.35 | 4.32 | 3.83 |
| 75 | 4.05 | 3.52 | 3.78 |
| 76 | 4.35 | 5.57 | 4.96 |
| 77 | 4.08 | 3.67 | 3.88 |
| 78 | 6.88 | 7.74 | 7.31 |
| 79 | 3.68 | 3.07 | 3.38 |
| 80 | 5.01 | 5.57 | 5.29 |
| 81 | 4.01 | 4.74 | 4.38 |
| 82 | 4.10 | 5.05 | 4.58 |
| 83 | 4.10 | 4.38 | 4.24 |
| 84 | 3.85 | 4.30 | 4.08 |
| 85 | 4.10 | 3.55 | 3.83 |
| 86 | 4.10 | 3.55 | 3.83 |
| **DH progeny No.** | **RS_2019** | **WS_2019-20** | **Mean disease score** |
| 87 | 5.10 | 4.05 | 4.58 |
| 88 | 4.85 | 4.71 | 4.78 |
| 89 | 3.30 | 4.55 | 3.93 |
| 90 | 4.10 | 4.71 | 4.41 |
| 91 | 5.10 | 5.30 | 5.20 |
| 92 | 5.35 | 6.05 | 5.70 |
| 93 | 5.60 | 6.71 | 6.16 |
| 94 | 4.10 | 4.55 | 4.33 |
